# Indirectly estimated absolute lung cancer mortality rates by smoking status and histological type based on a systematic review

**DOI:** 10.1186/1471-2407-13-189

**Published:** 2013-04-09

**Authors:** Peter N Lee, Barbara A Forey

**Affiliations:** 1 P N Lee Statistics and Computing Ltd, Sutton, Surrey, UK

**Keywords:** Lung cancer, Absolute rates, Squamous cell carcinoma, Adenocarcinoma, Smoking

## Abstract

**Background:**

National smoking-specific lung cancer mortality rates are unavailable, and studies presenting estimates are limited, particularly by histology. This hinders interpretation. We attempted to rectify this by deriving estimates indirectly, combining data from national rates and epidemiological studies.

**Methods:**

We estimated study-specific absolute mortality rates and variances by histology and smoking habit (never/ever/current/former) based on relative risk estimates derived from studies published in the 20^th^ century, coupled with WHO mortality data for age 70–74 for the relevant country and period. Studies with populations grossly unrepresentative nationally were excluded. 70–74 was chosen based on analyses of large cohort studies presenting rates by smoking and age. Variations by sex, period and region were assessed by meta-analysis and meta-regression.

**Results:**

148 studies provided estimates (Europe 59, America 54, China 22, other Asia 13), 54 providing estimates by histology (squamous cell carcinoma, adenocarcinoma). For all smoking habits and lung cancer types, mortality rates were higher in males, the excess less evident for never smokers. Never smoker rates were clearly highest in China, and showed some increasing time trend, particularly for adenocarcinoma. Ever smoker rates were higher in parts of Europe and America than in China, with the time trend very clear, especially for adenocarcinoma. Variations by time trend and continent were clear for current smokers (rates being higher in Europe and America than Asia), but less clear for former smokers. Models involving continent and trend explained much variability, but non-linearity was sometimes seen (with rates lower in 1991–99 than 1981–90), and there was regional variation within continent (with rates in Europe often high in UK and low in Scandinavia, and higher in North than South America).

**Conclusions:**

The indirect method may be questioned, because of variations in definition of smoking and lung cancer type in the epidemiological database, changes over time in diagnosis of lung cancer types, lack of national representativeness of some studies, and regional variation in smoking misclassification. However, the results seem consistent with the literature, and provide additional information on variability by time and region, including evidence of a rise in never smoker adenocarcinoma rates relative to squamous cell carcinoma rates.

## Background

Extensive data are available by age, sex, year and country on lung cancer mortality rates [1] and on the prevalence of smoking [2]. There are also a large number of epidemiological case-control and prospective studies which provide estimates of the relative risk of lung cancer by various aspects of smoking, a recent meta-analysis [3] having considered data from 287 studies published in the 1900s. However, mainly because smoking habits are not usually recorded on death certificates (and would perhaps be of dubious validity if they were), it is actually quite difficult to obtain national data on lung cancer mortality rates by smoking habit. There are some publications based on prospective studies which present evidence on variation in lung cancer rates in never smokers by time (e.g. [4-8]) or by age and sex (e.g. [8-15]), but these data are predominantly from the USA, often 20 years or more old, and sometimes based on very few deaths or cases. Data on rates in former and current smokers and by histological type are even more limited.

The lack of data on absolute risk of lung cancer by smoking habit is a serious deficiency as it limits interpretation of the evidence. For example, it is clear that the relative risk of lung cancer associated with smoking reported in studies in China is substantially less than that reported in North American and European studies [3]. However, this may be because, in China, lung cancer rates in never smokers are higher and in ever smokers similar to those in the West, or because rates in ever smokers are lower, rates in never smokers being similar. While these two possibilities (among others) imply different roles of smoking and non-smoking factors, one cannot readily distinguish them from the currently available evidence. Another example is the case of adenocarcinoma. It is apparent that rates of adenocarcinoma have been rising relative to squamous cell carcinoma, a change which has been linked to the type of cigarette smoked (e.g. [16]), but there seems to be no good evidence on whether rates of adenocarcinoma in never smokers have been rising over time, or stayed constant. Having evidence on this would seem crucial to the interpretation.

In this paper we use an indirect method for estimating absolute lung cancer mortality rates by smoking habit based on combining evidence from epidemiological studies of smoking and lung cancer and national data on lung cancer rates. This allows estimation of how mortality rates vary by sex, country and time period separately for never, former, current and ever smokers and separately for total lung cancer, squamous cell carcinoma and adenocarcinoma. While, as will be discussed, the indirect method has some limitations, the estimates derived should add useful insight into the evidence on smoking and lung cancer.

## Methods

### The indirect method

#### Overall lung cancer mortality rates

Suppose the population is divided into S + 1 smoking groups according to smoking habit, with i = 0 referencing never smokers and i = 1…S referencing subdivisions of ever smokers. For a case-control study, the data can be expressed in a 2 × (S+1) table, with N_1i_ referring to the number of cases and N_2i_ to the number of controls in smoking group i, and N_1_ and N_2_ to the total numbers of cases and controls respectively.

For smoking group i, define p_1i_ as the proportion of cases (= N_1i_ / N_1_), p_2i_ as the corresponding proportion of controls (= N_2i_ / N_2_), and R_i_ as the relative risk of lung cancer compared to never smokers.

Suppose that L_W_ is an estimate of the overall lung cancer rate in the population from which the study was drawn, based on a total of N_W_ cases. L_i_, the lung cancer rates by smoking group, can be estimated based on the following equations:

(1) $$R_{i}=\left(p_{1i}p_{20}\right)/\left(p_{10}p_{2i}\right)$$

(2) $$L_{i}=R_{i}L_{0}$$

(3) $$L_{w}=\sum_{j=0}^{\text{S}}\left(p_{2j}L_{j}\right)$$

These solve directly to give:

(4a) $$L_{i}=L_{w}p_{1i}/p_{2i}$$

or alternatively

(4b) $$L_{i}=L_{w}R_{i}/\sum_{j=0}^{S}\left(p_{2j}R_{j}\right)$$

The variance of the logarithm of the rate estimate, L_i_, can then be estimated approximately as:

(5) $$\begin{array}{l} \text{var}\text{log}L_{i}=\left(1/{\text{N}}_{w}\right)+\left(\left(1-p_{1i}\right)/{\text{N}}_{1}p_{1i}\right)\\ \;+\left(\left(1-p_{2i}\right)/{\text{N}}_{2}p_{2i}\right) \end{array}$$

The inverse of var log L_i_ can be used as a weighting factor in meta-analysis.

In the present work, the formulae are applied either to estimate lung cancer rates in never and ever smokers or to estimate lung cancer rates in never, former and current smokers.

In some studies observed counts may be zero. Here p_1i_, p_2i_ and R_i_ are estimated by adding 0.5 to each cell of the relevant 2 × (S + 1) table. While this approach is questionable, estimates derived in this way have very small weight, so contribute little to meta-analyses.

The method described above is based on data from case-control studies unadjusted for covariates. It is also applied to unadjusted data from prospective studies, with N_2_ and N_2i_ representing the numbers in the at risk population.

The method can also be applied where there is covariate adjustment, and the data available consist of the relative risks, the numbers of cases by smoking group, and the total number in the at risk population. Here p_2i_ is estimated by:

(6) $$p_{2i}=\left(p_{1i}/p_{10}R_{i}\right)/\sum_{j=0}^{\text{S}}\left({p_{1j}/p_{10}R}_{j}\right)$$

and formulae (4) and (5) then applied.

#### Lung cancer rates by histological type

Let z_h_ be the proportion of lung cancer with histological type h. The overall lung cancer rate for type h is then given by:

(7) $$L^{h}=z_{h}L_{w}$$

and $L_{i}^{h}$, the rates by smoking group for histological type h, are estimated using formulae corresponding to formulae (4a) and (4b) as:

(8a) $$L_{i}^{h}=L^{h}p_{1i}^{h}/p_{2i}^{h}$$

or alternatively as:

(8b) $$L_{i}^{h}=L^{h}R_{i}^{h}/\sum_{j=0}^{\text{S}}\left(p_{2i}^{h}R_{j}^{h}\right)$$

Here the superscript h implies that the proportions and relative risks are estimated from the set of cases and controls (or at risk) relating to the histological type. In some case-control studies, the controls are specific to the histological type, but in others they are common to all lung cancer cases.

Here the variance of the logarithm of the rates is estimated as:

(9) $$\begin{array}{c} \text{var}\text{log}L_{i}^{h}=\left(1/{\text{N}}_{w}\right)+\left(\left(1-z_{h}\right)/{\text{N}}_{1}z_{h}\right)\\ \;+\left(\left(1-p_{1i}^{h}\right)/{\text{N}}_{1}^{h}p_{1i}^{h}\right)\\ \;+\left(\left(1-p_{2i}^{h}\right)/{\text{N}}_{2}^{h}p_{2i}^{h}\right) \end{array}$$

Note that, in some studies, histological typing may only be carried out on a proportion of cases, the rest being classified as of unknown type. Here N_1_ in formula 9 should be replaced by the number of cases for which typing was carried out.

### Application of the method

To apply the indirect method, sex-specific data were extracted from the International Epidemiological Studies on Smoking and Lung Cancer (IESLC) database, which considers all epidemiological prospective and case-control studies involving over 100 lung cancer cases published in the last century, and has been described in detail elsewhere [3]. The data used relate to the relative risk of former, current and ever smoking, each relative to never smoking. For each study considered, the data extracted consisted of the components of the 2 × (S + 1) table and the relative risks, with the distribution of controls or at-risk estimated, if not available, using formula (6).

Where there was a choice, relative risks for smoking of any product were selected if available, or of cigarettes (or cigarettes only) if not, then selecting the widest available age and race group, and, for prospective studies, the longest follow-up. Current and ex smoking relative risks were constrained to match each other on these selection criteria, but not necessarily to match the ever smoking relative risk. Where relevant (e.g. when using relative risks for ever smoking any product and for current and ex cigarette smoking) separate versions of the 2 × 2 (never/ever) and 2 × 3 (never/ex/current) tables were used, and the indirect estimate of the never smoker rate that is reported is that based on the never/ever comparison.

For all lung cancer, we only considered unadjusted relative risks from case-control studies, and unadjusted or age-adjusted relative risks from prospective studies, as these were more directly relevant for comparison with national mortality rates. (Note that according to the data-entry protocol for prospective studies in IESLC, an unadjusted relative risk would not have been entered on the database if an equivalent age-adjusted relative risk was available.) However, due to the sparsity of available data, relative risks adjusted for other potential confounders were also accepted for squamous cell carcinoma and adenocarcinoma (preferring the least-adjusted estimates where there was a choice).

“All lung cancer” was defined (as previously, [3]) as including at least squamous cell carcinoma and adenocarcinoma, “squamous” as including at least squamous cell carcinoma but not adenocarcinoma, and “adeno” as including at least adenocarcinoma but not squamous cell carcinoma. Studies presenting results for squamous but not adeno, or vice versa, were excluded, as were studies where the proportion of cases for which typing was carried out could not be estimated, typically where results were available only for specific cell types.

Sex-specific estimates of L_W_, the overall lung cancer rate, were derived from the WHO mortality database [1]. This provides data by sex, single years and five year age groups for an extensive list of countries. For each epidemiological study, a year was estimated corresponding to the midpoint of the period of the case-control study or, for prospective studies, the survival-adjusted midpoint of the period of follow-up (as further explained in footnote a of Table 1). If there were no WHO mortality data corresponding to that year, data for a substitute year (within 20 years) were used as also shown in Table 1. Data were not available for India, South Africa, Taiwan, Turkey or Zimbabwe, so epidemiological data from these countries were not considered in our analyses. Table 1 also shows the few cases where data for substitute countries were used. Data from multi-country studies were also not considered.

**Table 1:** Substitute years and countries used

**Source of epidemiological data**		**Substitute data taken from WHO database**		
**Country**	**Years**^**a**^	**Country**^**b**^	**Year**^**c**^	**ICD codes**^**d**^
Brazil	1991	Brazil South	-	-
China	1978–1987	-	1988	
	1988 onwards	China, selected urban and rural areas	-	C028^e^
Finland	1944–1951	-	1952	-
Germany^f^	1936	West Germany	1952	-
Hungary	1953	-	1955	-
Poland	1956	-	1959	-
Uruguay	1991–1995	-	1990	-
USA	1941–1949	-	1950	-
UK	1948	-	1950	-

^a ^For case-control studies, this is the midpoint of the years of the study. For other studies, it is the midpoint of the years of the baseline phase, plus *f* × years of follow-up where the survival factor *f* is taken as 0.45, 0.425, 0.40, 0.375, 0.35, 0.325 or 0.30 for, respectively, follow-up periods of 1–10, 11–15, 16–20, 21–25, 26–30, 31–35 and 36–40 years. If the follow-up period differs by smoking status, the value relevant to ever smoking is used.

^b ^Dash indicates that the country for which WHO data were extracted is the same as the country from which the epidemiological data came.

^c ^Dash indicates that the year for which WHO data were extracted is the same as the year for which the epidemiological data were relevant.

^d ^Dash indicates that the ICD codes used are A050 for the 6^th^ and 7^th ^revisions, A051 for the 8^th^, B101 for the 9^th^ and C33–C34 for the 10^th^, corresponding throughout to malignant neoplasm of the trachea, bronchus and lung. ICD = International Classification of Diseases.

^e ^Additionally includes carcinoma in situ.

^f ^For post-war/pre-unification epidemiological data, WHO data were extracted for East or West Germany as appropriate to the area where the study was conducted. For 1991 onwards, WHO data for unified Germany were extracted.

Given that the estimates of L_W_ are of national rates, the indirect method may be inappropriate for an epidemiological study that is based on a special population or is conducted in an area of high risk. While it is clearly best if the population considered in the epidemiological study is nationally representative, it may still give some useful information if the study is conducted in a major town in the country. It was decided therefore to consider all epidemiological study data except where the population studied was grossly unrepresentative. Studies excluded were those of occupational groups with a known or possible lung cancer risk, specific races forming a minority of the population, or special groups with an increased mortality risk, such as persons with high coronary risk.

### Testing the validity of the method with respect to age

While the WHO mortality data are by 5 year age group, the epidemiological data are typically for the whole age range considered, though for some studies estimates are available for less broad age ranges. The question therefore arises as to the validity of applying estimates of the ratio L_i_/L_W_ based on data for a wide age range to overall estimates of L_W_ for a range of 5 year age groups. Given that the proportion of smokers among both cases and controls will vary by age, estimates of L_i_/L_W_ are also likely to vary by age. However, it seems reasonable to hope that, if one chooses an age group fairly typical of the average age of lung cancer cases, then L_i_/L_W_ based on the total data will be quite accurate for that age group.

To test this idea, an investigation was carried out using data from the million person American Cancer Society Cancer Prevention Study I (CPSI) prospective study starting in 1959 [9]. This gives lung cancer deaths and person years by age, sex and smoking status (never/former/current) for whites. The actual rate of lung cancer (per 100,000 per year) among never smokers by age was estimated and compared with that predicted based on the overall lung cancer rates by age and an estimate of L_0_/L_W_ derived from the total data ignoring age. Table 2 shows the results for ages 45–49 up to 85–89 for both sexes. As is evident, the predicted rate tends to be an overestimate for younger age groups and an underestimate for older age groups. However, it is reasonably accurate for age groups 65–69, 70–74 and 75–79. We reached similar conclusions based on data from the 1.25 million person US Cancer Prevention Study II prospective study starting in 1982 [15] (results not shown).

**Table 2:** **Lung cancer rates**^**a **^**in never smokers observed in CPSI**^**b **^**and predicted using the indirect method**

	**Males**			**Females**		
**Age**	**Lung cancers**	**Observed rate**	**Predicted rate**	**Lung cancers**	**Observed rate**	**Predicted rate**
45–49	2	2.62	5.54	14	3.69	7.12
50–54	10	6.87	10.02	30	5.01	9.80
55–59	22	11.82	17.65	49	6.94	11.05
60–64	29	17.41	29.49	95	14.39	17.32
65–69	41	31.41	38.67	92	16.78	20.05
70–74	32	33.42	44.28	86	21.01	19.79
75–79	32	52.30	47.88	100	38.39	30.76
80–84	26	85.99	41.21	63	47.58	33.35
85–89	17	48.61	41.51	35	67.05	47.19
Total	215	22.39	22.39	573	14.22	14.22

Note: L_0_/L_W _was estimated as 0.1695 for males, and 0.7008 for females.

^a ^mortality rates per 100,000 per year.

^b ^American Cancer Society Cancer Prevention Study I.

Overall, the correspondence between observed and predicted rates was best for age 70–74, and it was decided to use the epidemiological data to estimate L_i_/L_W_, and then apply it to the WHO national data for age 70–74. However we excluded from consideration epidemiological studies of young populations, where the upper age limit of the population studied was less than or equal to 60 years or where the age range of the population was unknown.

### Meta-analysis

Inverse-variance weighted fixed-effect and random-effects meta-analyses were conducted by standard methods [17], with heterogeneity quantified by H, the ratio of the heterogeneity chi-squared to its degrees of freedom, which is directly related to the statistic I^2^[18] by the formula I^2^ = 100(H - 1)/H. Meta-analyses were conducted separately for overall lung cancer rates and also for squamous and for adeno. Estimates were derived for total rates and for rates by the factors sex, region and grouped year of study. Tests of variation in rates by individual factor levels were carried out taking into account the extra-binomial variability of the data. Thus if H_0_ and D_0_ are the heterogeneity chi-squared values and degrees of freedom for the total data (based on a total of M estimates) and H_j_ and D_j_ are the corresponding values for each of m levels of the factor, the expression

$$\frac{\left(h_{0}-\sum h_{j}\right)/\left({\text{D}}_{0}-\sum {\text{D}}_{j}\right)}{\sum h_{j}/\sum {\text{D}}_{j}}$$

(where summation is over the m levels of the factor) can be considered an approximate F statistic on m-1, M-m degrees of freedom.

### Meta-regression

Inverse-variance weighted regression analyses were conducted, separately for males and females, to further assess the effects of region and time period. A continuous “linear period” variable was defined as 1 = 1930–60, 2 = 1961–70, 3 = 1971–80, 4 = 1981–90, 5 = 1991–99, and a categorical “continent” variable was defined to take the levels America, Europe, China and Asia (not China). Estimates were derived of the means and standard errors (SEs) for the model with both factors fitted, and the significances of linear period unadjusted for continent, continent unadjusted for linear period, linear period adjusted for continent and continent adjusted for linear period were tested. Additional analyses tested for the effects of introducing a fuller 10 level region variable (Canada, USA, South or Central America, UK, Scandinavia, West Europe, East Europe, Japan, China, Other Asia), the fuller 5 level period variable, or interactions between continent and linear period.

### Software

Analysis was carried out using ROELEE version 3.1 (available from P.N. Lee Statistics and Computing Ltd, 17 Cedar Road, Sutton, Surrey SM2 5DA, UK) and Excel 2003.

## Results

### Studies

Table 3 summarizes features of the 148 studies from 29 countries used for indirect estimation. Reasons for rejecting 139 studies are given in Additional file 1. The most common reasons for rejection were no relative risks available for ever vs never smokers (32 studies), only combined-sexes results available (45 studies), and study in an occupational group with a known or possible lung cancer risk (22 studies). Of the included studies, 7 were conducted in Canada, 40 in the USA, 7 elsewhere in the Americas, 17 in the UK, 13 in Scandinavia, 22 elsewhere in Western Europe, 7 in Eastern Europe, 9 in Japan, 22 in China (including Hong Kong), and 4 elsewhere in Asia. There were 120 case-control studies, 25 prospective studies, two of nested case-control and one of case-cohort design. 78 of the studies provided results for both sexes, 54 for males only, and 16 for females only. 144 provided results for total lung cancer, and 54 for squamous and adeno.

**Table 3:** Epidemiological studies used for indirect estimates

**Region / Country**^**a**^	**Study**^**b**^	**Study design**^**c**^	**Year**^**d**^	**Race**^**e**^	**Sex**^**f**^	**Smoking status**^**g**^	**Product**^**h**^	**Lung cancer type**^**i**^
**Canada**	BAND^j^	CC	1987	all	m	E	Conly(1)	q, a
	BEST	P	1957	all	m	E,	A	all
						C,X	Conly(1)	all
			1958		f	E	Conly(1)	all
	HOROWI	CC	1962	all	m, f	E	C(1)	all
	JAIN	CC	1983	all	m, f	E,C,X	C	all, q, a
	MCDUFF	CC	1981	all	m	E	C	all
	SIEMIA	CC	1982	all	m	E	C	all, q, a
	WIGLE	CC	1972	all	m, f	E,C,X	A	all
**USA**	ANDERS	P	1990	all	f	E,C,X	C	all,
						E	C	q, a
	BLOT4	CC	1976	wh	m	E	C	all
	BOUCOT	P	1958	all	m	E,	A	all
						C,X	Conly(1)	all
	BRESLO	CC	1951	all	m, f	E	A(2)	all
	BROSS	CC	1963	wh	m	E,	A	all
						C,X	C(1)	all
	BROWN2^j^	CC	1987	wh	m, f	E,C,X	C	q, a
	BUFFLE	CC	1978	wh	m	E	A	all
						E	C	q, a
						C,X	C(1)	all
				wh	f	E	A	all
				wh-hi		E	C	q
				wh		E	C	a
				wh		C,X	C(1)	all
				wh-hi		C,X	C(1)	q, a
	BYERS1^k^	CC	1961	wh	m	E	C	q, a
	CHANG	P	1980	all	m, f	E,C,X	C	all
	CHOW	P	1974	wh	m	E,C,X	A	all
	COMSTO	NCC	1987	all	m, f	E	A	all
						C,X	C(1)	all
						E,C,X	C	q, a
	CPSI	P	1962	all	m	E,	C(1)	all
				wh		C,X	Conly(1)	
				all	f	E,C,X	C	all
	CPSII	P	1984	all	m	E,C,X	Conly(1)	all
					f	E,C,X	C	all
	DORGAN	CC	1982	wh	m, f	E	A	all
				wh	m	E	C(1)	q, a
				all	f	E	C(1)	q, a
				wh	m, f	C,X	C(1)	all
	DORN	P	1959	wh	m	E,C,X	A	all
	GOODMA	CC	1984	w + o	m, f	E,C,X	C(1)	all
	GRAHAM	CC	1958	wh	m	E,C,X	A	all
	HAENSZ	CC	1956	all	f	E	A	not alv, q + u, a
						C,X	C(1)	not alv, q + u, a
	HAMMON	P	1953	wh	m	E	A	all^l^, not a, a
	HENNEK	P	1988	all	m	E,C,X	A	all
	HORWIT	CC	1980	all	f	E	C	all
	KAISE2	P	1987	all	m, f	E,C,X	Conly(1)	all
	KELLER	CC	1986	wh	m, f	E,C,X	A	all
	KHUDER	CC	1986	all	m	E,C,X	C	all, q, a
	LOMBA2	CC	1964	all	f	E	C	all, q + u, not q + u
	LOMBAR	CC	1958	all	m	E	A	all
						C,X	C(1)	all
	MILLER	CC	1978	all	f	E	C(1)	all
	NAM	CC	1986	all	m, f	E,C,X	C	all
	OSANN	CC	1985	all	m, f	E,C,X	C	all, q, a
	OSANN2^k^	NCC	1973	all	f	E,C,X	C	KI, KII
	PIKE	CC	1974	w-hi	m, f	E	A	all
	SADOWS	CC	1941	wh	m	E	A	all
	SCHWAR	CC	1986	wh	m, f	E,C,X	C	all
	STAYNE	CC	1970	all	m	E	A	all, q, a
	TOUSEY	CC	1995	all	m, f	E,	A	all
						C,X	C(1)	all
	WU	CC	1982	wh	f	E,C,X	A	q + a
	WYNDE2	CC	1963	all	m	E	A	all, KI, KII
	WYNDE3	CC	1968	all	m	E,C,X	A	all, KI, KII
					f	E	A	all, KI, KII
	WYNDE4	CC	1949	all	m	E	A	all, not a, a
					f ^j^	E	A	not a, a
	WYNDE6	CC	1983	all	m	E,	A	all, KI, KII
						C,X	C(1)	all, KI, KII
				all	f	E,C,X	C	all
				wh		E	C	q, a
				all		C,X	C	KI, KII
**SC America**								
Uruguay	DESTE2	CC	1995	all	m	E	A	all, q, a
Uruguay	DESTEF	CC	1991	all	m	E,C,X	A	all
Cuba	JOLY	CC	1979	all	m	E,C,X	A	all
						E	C(1)	q, a
					f	E,C,X	C(1)	all
						E	C(1)	q, a
Argentina	MATOS	CC	1995	all	m	E,C,X	C(1)	all, q, a
Argentina	PEZZO2	CC	1995	all	m	E,C,X	C	all
Argentina	PEZZOT	CC	1989	all	m	E,C,X	Conly	all
						E	Conly	q, a
Brazil	WUNSCH	CC	1991	all	m, f	E,C,X	C(1)	all
**UK**	ALDERS	CC	1980	all	m	E	A	all^l^, q, a
					f	E^l^	MConly(1)	all
						E	A	q, a
	BENSHL	P	1973	all	m	E,C,X	A	all
	BRETT	P	1961	all	m	E,C,X	C	all
	DARBY	CC	1991	wh	m, f	E^m^	A	all
	DEAN2	CC	1961	all	m, f	E,C,X	A	all
	DEAN3	CC	1971	all	m	E,C,X	A	all
					f	E,C,X	MConly(1)	all
	DOLL	CC	1950	all	m, f	E,C,X	A	all
						E	A	KI, KII
	DOLL2	P	1963	all	m	E,C,X	A	all
	GILLIS	CC	1979	all	m	E,C,X	C(1)	all
	GOLLED	CC	1957	all	m	E	C(1)	all
	GREGOR	CC	1977	all	m, f	E,C,X	C	all
	HOLE	P	1979	all	m	E,C,X	A	all
	MCCONN	CC	1948	all	m, f	E	A	all
	MIGRAN	P	1970	all	m, f	E,C,X	A	all
	PETO	P	1966	all	m	E,C,X	A	all
	STOCKS	CC	1954	all	m	E	A	all
	WILKIN	CC	1993	all	m, f	E	C	all
**Scandinavia**								
Sweden	AXELSS	CC	1991	sca	m, f	E,C,X	A	all
Sweden	DAMBER	CC	1975	all	m	E	A	all, q, a + al + br
Norway	ENGELA	P	1970	all	m	E	A	all, q, a
						C,X	C	all, q, a
					f	E	A	all
						C,X	C	all
Norway	KJUUS	CC	1981	all	m	E,C,X	A	all
Finland	KNEKT	P	1977	all	m	E,C,X	A	all
Finland	KOULUM	CC	1944	all	m	E	A	all
Norway	KREYBE	CC	1951	all	m, f	E	A	all, KI, KII
Denmark	LANGE	P	1982	all	m, f	E,C,X	A	all
Sweden	NOU	CC	1974	all	m, f	E	A	all, q, a
Finland	PERNU	CC	1951	all	m, f	E	A	all
Sweden	SVENSS	CC	1985	all	f	E,C,X	A	all, q, a
Finland	TENKAN	P	1969	all	m	E,C,X	A	all
Iceland	TULINI	P	1985	all	m, f	E,C,X	A	all
**W Europe**								
Switzerland	ABELIN	CC	1953	all	m	E	A	all
Spain	AGUDO	CC	1991	all	f	E,C,X	Conly(1)	all
Spain	ARMADA	CC	1988	all	m	E	A	all
						C,X	C(1)	all
Italy	BARBON	CC	1983	all	m	E,C,X	A	all, q, a
Germany	BECHER	CC	1986	all	m, f	E,C,X	A	all
					f	E	A	q + s, not q + s
France	BENHAM	CC	1978	all	m	E	A	all
						C,X	Conly(1)	all-mix
						E,C,X	Conly(1)	KI, KII
					f	E	C(1)	all, KI, KII
Germany	BLOHMK	CC	1979	all	m	E,C,X	A	all
Germany	BROCKM	CC	1991	wh	m, f	E	C	all
Germany	DAVEYS	CC	1936	all	m, f	E	A	all
Netherlands	DORANT	CCO	1987	all	m	E,C,X	A	all
Belgium	DROSTE	CC	1996	all	m	E,C,X	A	all
Germany	EBELIN	CC	1983	all	m	E	A	all
Switzerland	GSELL	CC	1946	all	m	E	A	all
Germany	JAHN	CC	1991	all	m	E,	A	all, q, a
						C,X	C(1)	all, q, a
					f	E	C(1)	all
Greece	KATSOU	CC	1988	all	f	E,C,X	A	all, KI, a
Germany	KREUZE	CC	1993	all	m, f	E,C,X	A	all
Italy	PASTOR	CC	1978	all	m	E	A	all
Germany	RANDIG	CC	1953	all	m, f	E	A	all
Italy	RONCO	CC	1978	all	m	E	A	all
France	STUCKE	CC	1991	all	m	E,C,X	A	all
Italy	TIZZAN	CC	1960	all	m, f	E,C,X	A	all
Austria	VUTUC	CC	1978	all	m	E^n^	C	all, KI, KII
					f	E,C,X	C	all, KI, KII
**E Europe**								
Hungary	ABRAHA	P	1984	all	m, f	E	A	q + s + a, q, a
Poland	JEDRYC	CC	1984	all	m, f	E^o^	C(1)	all,
					m	E,C,X	C(1)	q, a
Czechoslovakia	KUBIK	P	1968	all	m	E	A	all
						C,X	C(1)	all
Hungary	ORMOS	CC	1953	all	m	E	C(1)	all, q, a
					f	E	C(1)	all
Poland	PAWLEG	CC	1993	all	m	E	A	all
Poland	RACHTA	CC	1993	all	f	E,C,X	C	all
Poland	STASZE	CC	1956	all	m, f	E	A	all, q, a
**Japan**	ESAKI	CC	1966	all	m, f	E	C	all
	GAO2	CC	1990	all	m	E,C,X	C	all
	HIRAYA	P	1972	all	m, f	E,C,X	C(1)	all
	HITOSU	CC	1963	all	m, f	E,C,X	A	all
	KIHARA	CC	1995	jap	m	E	A	all
	MATSUD	CC	1965	all	m	E	C	all, q, a
	SEGI	CC	1950	all	m	E	A	all
	SOBUE	CC	1987	all	m, f	E,C,X	C	q + s + l + a, q, a
	WAKAI	CC	1990	all	m, f	E,C,X	A	all, q, a
**China**								
Hong Kong	CHAN	CC	1977	all	m, f	E	A	all, q + s, a + l
China	CHEN2	CC	1983	all	m, f	E	A	all
China	DU	CC	1985	all	m, f	E	A	all
China	FAN	CC	1991	all	m, f	E	C(1)	all
China	GAO	CC	1985	all	m, f	E,C,X	C	all
						E	C	q, a
China	GENG	CC	1988	all	m, f	E	C(1)	all
China	HU	CC	1986	all	m, f	E	C(1)	all
China	HU2	CC	1978	all	m, f	E	C	all
China	JIANG	CC	1984	all	m, f	E	A	all
Hong Kong	KOO	CC	1982	all	f	E,C,X^p^	A	all
						E	A	q + s, a + l
Hong Kong	LAMTH	CC	1985	ch	f	E	A	all, q, a
Hong Kong	LAMWK	CC	1983	ch	f	E	A	all, q, a
Hong Kong	LAMWK2	CC	1978	all	m, f	E	A	q + s + l + a, q, a
China	LEI	CC	1986	all	m, f	E	A	all
China	LIU2	CC	1984	all	m, f	E	A	all
China	LIU3	CC	1986	all	m	E	A	all
China	LIU4	CC	1987	all	m, f	E	A	all
China	WANG	CC	1992	all	m, f	E	A	all
China	WUWILL	CC	1986	all	f	E	C	all, q, a
China	XU	CC	1986	all	m	E	A	all
China	XU3	CC	1981	all	m, f	E	A	all, KI, KII
China	ZHOU	CC	1986	all	m, f	E	A	all, q, a
**Other**								
S Korea	CHOI	CC	1987	all	m, f	E,C,X	C	all
						E	C	q, a
Singapore	MACLEN	CC	1973	ch	m, f	E,C,X	C	all
Singapore	SEOW	CC	1998	ch	f	E	C	q + s + l + a, q, a
Thailand	SIMARA	CC	1972	all	m, f	E	C	all

^a ^Country not shown if same as region.

^b ^Six character reference codes used in IESLC. See Table two of [3] for associated reference(s).

^c ^CC = case-control, CCO = case-cohort, NCC = nested case-control, P = prospective.

^d ^See footnote a of Table 1.

^e ^ch = Chinese, jap = Japanese, o = oriental, sca = Scandinavian, wh = white, wh-hi = white excluding hispanic.

^f ^m = male, f = female.

^g ^E = ever vs never, C = Current vs never, X = Ex vs never. Studies with no ever vs never relative risk were excluded (see Additional file 1). Except where indicated below by footnotes l-o, studies shown only as “E” had no current vs never or ex vs never relative risk.

^h ^A = any product, C = cigarettes, MC = manufactured cigarettes. The comparison is between “ever smoked the product” and “never smoked the product” except where indicated (1) the comparison is with never smokers of any product (i.e. never smokers excluded pipe/cigar only smokers), (2) never smokers included long term ex smokers.

^i ^Indicates lung cancer types for which results are available, a = adenocarcinoma, all = total lung cancer, alv = alveolar, br = bronchioalveolar, KI = Kreyberg I, KII = Kreyberg II, l = large cell carcinoma, mix = mixed, q = squamous cell carcinoma, s = small or oat cell carcinoma, u = undifferentiated. Where only one entry is shown, results are only available for a definition of all lung cancer. Where three entries are shown, the first entry relates to the definition of all lung cancer, the second to the definition of squamous and the third to the definition of adeno. Where two entries are shown, the two entries relate to the definitions of squamous and adeno, no results being available for a definition of all lung cancer (as further explained in footnotes j and k).

^j ^All lung cancer not included as only adjusted relative risks available.

^k ^Subsidiary study, results for all lung cancer available from corresponding principal study.

^l ^Current smoking excluded because no ex smoking relative risk available.

^m ^Current and ex smoking excluded because no matching pair of relative risks available.

^n ^Current and ex smoking excluded because only available relative risks did not satisfy age criteria.

^o ^Ex smoking excluded because no current smoking relative risk available.

^p ^Current and Ex based on a subset of the study.

### Estimates

The indirect estimates of the lung cancer rates (per 100,000 per year) and their weights, by smoking habit, location and study, are given for total lung cancer in Table 4 (males) and Table 5 (females), for squamous in Table 6 (males) and Table 7 (females), and for adeno in Table 8 (males) and Table 9 (females). With some exceptions, the rates are lowest in never smokers, intermediate in former smokers and highest in current smokers, consistent with the general pattern of relative risks.

**Table 4:** **Indirect estimates of mortality rates**^**a **^**by smoking habit - all lung cancer, males**

			**Never smoked**		**Former smoker**		**Current smoker**		**Ever smoker**	
**Region**	**Country**^**b**^	**Study**^**c**^	**Rate**	**Weight**	**Rate**	**Weight**	**Rate**	**Weight**	**Rate**	**Weight**
Canada		BEST	6.2	1.0	70.8	17.6	174.2	252.7	156.6	266.2
		HOROWI	77.8	18.6					278.1	300.7
		JAIN	59.1	11.0	331.9	103.2	940.7	129.6	591.2	611.6
		MCDUFF	83.8	5.3					513.5	407.8
		SIEMIA	40.3	11.9					642.0	856.2
		WIGLE	43.6	14.2	309.7	108.2	516.2	358.5	444.8	626.4
USA		BLOT4	38.8	7.7					563.0	808.7
		BOUCOT	5.0	0.5	93.4	9.2	253.2	702.3	202.2	3203.4
		BRESLO	17.7	6.1					114.1	1284.3
		BROSS	55.7	33.0	325.1	109.0	296.3	582.3	286.8	2219.5
		BUFFLE	46.8	4.6	535.2	143.8	474.2	249.1	493.3	2886.8
		CHANG	109.5	5.3	435.1	85.4	918.7	54.1	569.6	985.3
		CHOW	47.3	6.2	193.2	31.1	688.1	911.6	504.0	4110.0
		COMSTO	55.4	3.9	328.5	50.6	967.4	102.3	599.1	882.9
		CPSI	32.2	85.2	111.4	335.8	364.3	4374.2	295.9	4642.6
		CPSII	52.5	83.4	470.6	1748.1	1124.2	1982.6	673.5	11885.8
		DORGAN	55.2	13.5	325.4	191.2	865.7	213.6	542.7	2113.1
		DORN	35.0	80.7	142.8	268.2	285.2	2347.6	246.3	3553.1
		GOODMA	62.4	10.1	368.6	76.7	1087.0	150.6	673.7	901.0
		GRAHAM	26.4	17.6	427.0	94.3	177.8	1078.2	204.7	2385.0
		HAMMON	22.5	15.4					145.0	1863.0
		HENNEK	128.9	26.5	472.8	109.4	1983.2	139.5	804.2	962.1
		KAISE2	143.0	16.5	448.6	36.0	1063.9	122.0	772.5	506.6
		KELLER	62.6	267.2	453.0	1313.5	824.9	1455.2	631.8	5420.3
		KHUDER	79.1	22.8	594.7	203.6	642.4	316.8	621.1	1889.8
		LOMBAR	21.7	12.7	114.7	84.0	218.8	1040.2	192.2	2474.5
		NAM	67.3	30.2	885.6	378.3	477.2	268.6	661.8	2244.7
		OSANN	40.5	45.3	497.7	255.7	1095.0	564.4	815.1	1838.5
		PIKE	86.2	15.1					452.9	1709.4
		SADOWS	28.1	15.4					108.6	1047.1
		SCHWAR	74.7	99.5	350.7	674.0	1116.9	406.9	613.1	2889.0
		STAYNE	134.4	59.2					492.8	862.3
		TOUSEY	27.7	4.0	373.0	177.5	1469.6	83.3	612.3	1602.8
		WYNDE2	33.4	7.6					281.8	2151.2
		WYNDE3	52.7	8.6	198.0	43.4	565.3	288.7	427.0	1141.2
		WYNDE4	12.4	11.1					112.8	1139.8
		WYNDE6	59.7	80.3	411.1	854.2	903.0	799.7	598.7	5040.3
SC America	Uruguay	DESTE2	85.1	24.6					570.0	137.9
	Uruguay	DESTEF	68.3	22.1	412.5	48.7	660.7	108.4	580.5	139.7
	Cuba	JOLY	35.7	11.4	314.2	72.4	475.2	275.5	434.8	347.2
	Argentina	MATOS	50.2	10.7	252.7	75.7	426.0	100.6	333.5	437.2
	Argentina	PEZZO2	20.8	5.8	191.7	123.8	477.6	169.1	312.4	678.0
	Argentina	PEZZOT	18.2	4.0	185.4	68.4	593.6	113.7	351.5	489.7
	Brazil	WUNSCH	47.2	12.9	165.4	92.9	267.1	187.5	219.6	657.6
UK		ALDERS	74.8	13.9					784.3	2386.1
		BENSHL	134.1	6.2	395.6	51.5	1096.9	547.8	791.3	2968.5
		BRETT	149.1	6.2	139.3	9.5	585.1	926.6	487.6	1638.2
		DARBY	13.1	3.0					648.1	3292.2
		DEAN2	131.4	27.0	496.6	51.5	498.6	1009.9	498.4	1703.2
		DEAN3	138.4	24.9	602.7	84.7	866.5	1286.8	812.9	2832.9
		DOLL	20.4	6.3	100.3	46.1	194.1	1011.7	185.1	1154.3
		DOLL2	73.3	19.1	305.8	266.4	805.8	1117.6	561.6	2915.3
		GILLIS	117.0	12.3	522.1	128.0	829.5	870.5	733.5	3445.4
		GOLLED	67.8	15.1					427.8	1623.4
		GREGOR	655.4	6.6	469.0	22.4	848.3	54.6	674.1	319.6
		HOLE	141.9	7.2	333.3	27.0	951.4	808.6	768.5	2647.8
		MCCONN	148.1	3.7					179.7	552.6
		MIGRAN	111.1	4.1	367.3	26.6	852.1	1004.6	738.5	3227.9
		PETO	98.3	2.0	75.0	2.0	705.3	1288.0	604.7	2008.3
		STOCKS	51.6	42.0					297.1	1793.5
		WILKIN	25.3	2.0					636.4	1166.6
Scandinavia	Sweden	AXELSS	36.6	15.2	167.7	82.5	546.5	99.1	310.9	279.6
	Sweden	DAMBER	45.6	35.7					333.0	251.1
	Norway	ENGELA	39.1	6.9	63.4	10.7	225.9	80.2	180.2	87.5
	Norway	KJUUS	24.2	1.9	151.0	30.9	509.1	72.7	332.4	176.1
	Finland	KNEKT	121.3	6.2	423.4	20.0	917.7	196.4	771.6	299.4
	Finland	KOULUM	8.9	4.4					315.2	72.9
	Norway	KREYBE	3.3	3.6					25.5	9.0
	Denmark	LANGE	117.3	5.0	283.5	22.0	648.7	343.6	569.7	445.7
	Sweden	NOU	48.9	6.1					297.8	184.2
	Finland	PERNU	44.3	40.2					395.6	71.9
	Finland	TENKAN	46.9	5.0	212.1	23.1	886.8	199.1	687.1	224.2
	Iceland	TULINI	42.4	4.3	120.0	6.4	440.8	8.0	341.8	8.0
W Europe	Switzerland	ABELIN	9.1	2.0					284.0	99.8
	Spain	ARMADA	22.4	3.8	347.6	104.2	548.0	128.5	441.5	757.4
	Italy	BARBON	50.5	20.7	359.9	120.3	669.9	469.3	557.8	1394.7
	Germany	BECHER	45.5	2.9	296.8	44.6	678.5	123.6	492.4	840.7
	France	BENHAM	32.7	34.9	312.7	235.6	492.7	782.3	406.9	2071.8
	Germany	BLOHMK	188.4	108.5	563.3	221.1	602.4	282.5	584.2	1026.2
	Germany	BROCKM	375.0	1.0					402.8	1208.1
	Germany	DAVEYS	26.7	2.8					127.9	369.3
	Netherlands	DORANT	101.4	6.8	436.4	161.4	1097.6	400.8	750.3	1217.4
	Belgium	DROSTE	51.9	6.6	360.4	74.6	978.7	308.1	733.1	804.9
	Germany	EBELIN	105.1	12.5					728.9	196.1
	Switzerland	GSELL	10.5	1.9					185.4	77.4
	Germany	JAHN	52.4	16.5	446.3	398.5	516.0	228.2	470.9	1877.1
	Germany	KREUZE	23.9	22.2	218.2	389.7	1002.0	565.0	520.7	2549.4
	Italy	PASTOR	72.0	9.6					474.7	656.7
	Germany	RANDIG	26.8	4.1					134.5	699.6
	Italy	RONCO	87.3	5.9					445.3	733.7
	France	STUCKE	3.3	0.5	451.4	183.5	347.7	46.4	416.8	725.4
	Italy	TIZZAN	54.6	110.5	109.5	153.1	103.4	305.4	105.3	439.2
	Austria	VUTUC	84.7	18.0					591.0	454.1
E Europe	Hungary	ABRAHA	69.5	10.2					607.9	755.4
	Poland	JEDRYC	92.3	43.0					536.1	1022.1
	Czechoslovakia	KUBIK	28.9	2.0	326.3	8.5	937.3	478.6	813.3	776.8
	Hungary	ORMOS	21.9	7.1					204.4	113.0
	Poland	PAWLEG	44.0	4.0					699.6	597.2
	Poland	STASZE	10.9	4.8					116.0	171.5
Japan		ESAKI	75.8	11.4					143.7	375.7
		GAO2	69.7	11.4	234.2	71.1	472.3	140.8	357.4	791.2
		HIRAYA	46.7	88.6	80.2	16.4	208.6	1645.6	203.5	1675.5
		HITOSU	43.9	7.1	199.8	25.5	111.2	315.2	120.7	624.6
		KIHARA	69.9	20.4					413.8	531.1
		MATSUD	7.5	3.0					160.5	797.2
		SEGI	12.9	16.5					22.0	99.9
		SOBUE	83.2	28.1	247.5	215.4	354.5	769.2	316.1	2720.6
		WAKAI	92.2	9.1	231.2	50.6	382.0	319.2	332.2	1426.3
China	HK	CHAN	18.3	1.9					504.0	88.1
	China	CHEN2	76.7	7.9					350.7	274.8
	China	DU	89.6	22.7					316.3	1155.0
	China	FAN	148.0	37.0					420.8	619.4
	China	GAO	89.5	53.1	344.9	76.1	352.0	514.8	350.5	950.9
	China	GENG	63.5	6.4					380.3	171.7
	China	HU	172.0	36.6					358.9	142.9
	China	HU2	119.8	42.2					362.5	415.7
	China	JIANG	115.7	5.5					315.0	307.7
	HK	LAMWK2	200.4	15.2					567.8	105.5
	China	LEI	93.7	34.7					345.0	743.8
	China	LIU2	76.7	10.2					331.1	557.7
	China	LIU3	236.7	3.6					285.2	447.1
	China	LIU4	129.9	1402.0					373.4	2121.6
	China	WANG	131.8	24.8					457.5	504.6
	China	XU	131.3	84.4					345.1	823.1
	China	XU3	63.5	6.4					380.3	171.7
	China	ZHOU	162.9	55.9					384.9	81.8
Other	S Korea	CHOI	37.3	11.7	70.1	29.3	192.1	178.9	156.4	241.8
	Singapore	MACLEN	78.1	3.5	106.5	3.2	297.3	25.4	279.7	26.2
	Thailand	SIMARA	7.0	17.2					13.9	18.9

^a ^Mortality rates per 100,000 per year for age 70–74 years.

^b ^Not shown if same as region. HK = Hong Kong.

^c ^Six character reference codes used in IESLC. See Table two of [3] for associated reference(s).

**Table 5:** **Indirect estimates of mortality rates**^**a **^**by smoking habit – all lung cancer, females**

			**Never smoked**		**Former smoker**		**Current smoker**		**Ever smoker**	
**Region**	**Country**^**b**^	**Study**^**c**^	**Rate**	**Weight**	**Rate**	**Weight**	**Rate**	**Weight**	**Rate**	**Weight**
Canada		BEST	17.6	17.7					39.5	3.8
		HOROWI	23.7	17.1					43.1	28.4
		JAIN	23.0	45.9	82.8	50.1	291.2	88.7	188.1	180.0
		WIGLE	24.8	36.6	50.5	9.7	121.9	55.8	101.9	70.4
USA		ANDERS	39.2	52.8	257.6	111.4	854.9	517.1	513.9	1754.5
		BRESLO	22.6	13.0					31.2	11.1
		BUFFLE	18.5	39.2	93.9	64.9	153.1	211.5	132.0	506.0
		CHANG	44.0	13.7	94.4	15.2	226.9	62.8	161.9	178.6
		COMSTO	37.2	14.1	103.5	9.5	487.8	57.1	333.2	122.8
		CPSI	17.9	232.9	24.3	15.4	56.3	195.3	49.8	223.1
		CPSII	38.2	204.8	184.6	338.5	471.5	975.6	311.4	2503.8
		DORGAN	27.3	93.2	86.0	77.7	332.7	143.5	214.2	382.5
		GOODMA	39.1	22.4	239.5	17.1	377.5	46.4	324.4	102.3
		HAENSZ	19.7	112.2	32.3	3.3	42.7	57.5	42.4	65.8
		HORWIT	18.9	11.7					213.4	135.1
		KAISE2	35.2	12.8	166.7	17.0	480.9	166.4	355.1	456.5
		KELLER	28.4	440.9	258.0	383.3	412.5	829.7	354.4	1571.4
		LOMBA2	25.9	81.1					34.4	220.5
		MILLER	20.5	33.2					232.3	515.7
		NAM	37.9	59.3	391.3	151.1	366.5	120.4	379.6	512.5
		OSANN	26.3	103.3	214.0	94.4	487.3	363.9	392.2	675.2
		PIKE	21.7	35.4					105.0	136.3
		SCHWAR	33.3	182.4	153.4	201.1	520.5	330.3	331.5	885.6
		TOUSEY	27.7	13.5	233.6	63.4	784.1	73.4	434.2	355.1
		WU	39.7	29.2	62.1	22.8	258.2	90.4	173.9	237.2
		WYNDE3	22.1	24.2					69.0	56.6
		WYNDE6	25.2	157.9	138.8	201.4	369.2	397.7	262.4	960.3
SC America	Cuba	JOLY	37.0	39.6	253.8	14.5	277.1	48.9	272.0	60.1
	Brazil	WUNSCH	23.6	35.9	78.0	13.4	136.1	31.5	103.8	64.8
UK		ALDERS	38.0	67.7					175.9	526.8
		DARBY	28.0	24.0					343.2	642.9
		DEAN2	36.7	120.7	126.2	1.5	106.3	24.2	107.7	26.6
		DEAN3	43.6	52.6	43.7	7.1	144.0	215.4	125.5	259.5
		DOLL	25.1	37.9	46.3	4.8	52.3	38.4	51.3	51.2
		GREGOR	13.2	1.0	72.5	4.0	189.6	27.1	145.0	90.1
		MCCONN	26.9	7.7					74.0	2.2
		MIGRAN	18.3	4.5	28.0	1.0	150.7	166.6	132.0	204.7
		WILKIN	53.9	12.6					308.2	167.2
Scandinavia	Sweden	AXELSS	17.5	17.6	52.3	11.0	207.8	51.1	150.7	77.3
	Norway	ENGELA	15.7	11.0	27.7	5.0	548.1	9.1	51.4	10.9
	Norway	KREYBE	24.1	9.9					19.3	6.6
	Denmark	LANGE	36.8	7.3	83.4	8.4	135.0	78.5	124.9	97.9
	Sweden	NOU	17.9	5.2					127.2	20.7
	Finland	PERNU	45.1	23.2					84.9	10.3
	Sweden	SVENSS	15.2	28.7	39.9	16.3	128.3	38.4	92.5	58.1
	Iceland	TULINI	16.7	3.4	62.5	3.6	318.0	5.0	249.6	5.0
W Europe	Spain	AGUDO	25.2	127.5	28.8	2.0	67.8	10.1	57.6	13.0
	Germany	BECHER	20.9	11.2	30.2	4.4	137.8	25.9	93.8	52.8
	France	BENHAM	17.5	73.4					77.8	25.4
	Germany	BROCKM	34.7	3.9					69.5	86.5
	Germany	DAVEYS	28.8	144.4					20.8	0.4
	Germany	JAHN	32.9	55.8					101.6	78.6
	Greece	KATSOU	34.7	41.4	96.8	2.8	120.9	15.1	116.5	19.0
	Germany	KREUZE	32.6	100.2	45.3	22.9	191.4	54.9	123.4	116.5
	Germany	RANDIG	21.1	27.6					46.9	18.3
	Italy	TIZZAN	18.1	38.2	66.1	4.9	78.1	10.9	73.8	18.8
	Austria	VUTUC	31.4	74.7	161.2	26.5	245.7	40.5	209.5	63.4
E Europe	Hungary	ABRAHA	37.0	34.2					180.6	102.0
	Poland	JEDRYC	27.8	88.9					222.0	31.3
	Hungary	ORMOS	38.4	42.1					7.4	1.0
	Poland	RACHTA	29.3	37.0	112.9	6.1	189.5	32.6	171.7	46.4
	Poland	STASZE	13.0	25.8					56.2	6.9
Japan		ESAKI	29.6	53.7					72.8	17.4
		HIRAYA	42.0	436.0	125.0	4.0	98.2	101.1	99.0	105.8
		HITOSU	19.6	52.8	164.6	5.6	68.6	38.5	76.5	51.2
		SOBUE	49.3	283.7	126.5	23.1	143.1	77.8	138.5	116.9
		WAKAI	47.8	95.3	138.6	2.6	175.9	19.1	169.9	23.7
China	HK	CHAN	106.9	47.9					371.5	32.0
	China	CHEN2	88.2	25.3					147.4	34.7
	China	DU	78.5	82.9					151.6	169.0
	China	FAN	79.6	107.9					312.1	70.7
	China	GAO	91.5	491.8	284.3	21.6	216.4	71.6	232.1	100.1
	China	GENG	66.1	71.6					195.6	84.5
	China	HU	97.0	60.8					168.1	15.5
	China	HU2	83.8	73.0					157.1	86.4
	China	JIANG	85.8	22.2					213.4	5.6
	HK	KOO	114.7	55.0	392.9	7.2	300.0	17.6	317.5	41.1
	HK	LAMTH	129.0	94.8					491.3	61.7
	HK	LAMWK	117.5	58.8					484.0	30.8
	HK	LAMWK2	119.8	46.5					384.1	31.0
	China	LEI	67.3	101.2					234.7	63.1
	China	LIU2	64.1	50.0					273.3	24.3
	China	LIU4	81.7	994.6					359.5	761.3
	China	WANG	115.4	303.6					461.7	4.1
	China	WUWILL	80.4	345.3					178.0	282.6
	China	XU3	60.5	16.1					243.4	12.5
	China	ZHOU	95.5	125.7					211.8	8.1
Other	S Korea	CHOI	32.4	80.7	140.0	2.0	39.6	8.8	51.2	11.9
	Singapore	MACLEN	40.4	6.3	32.2	1.8	95.9	5.9	84.7	6.1
	Singapore	SEOW	90.8	35.2					501.6	10.8
	Thailand	SIMARA	1.7	7.5					4.0	5.6

^a ^Mortality rates per 100,000 per year for age 70–74 years.

^b ^Not shown if same as region. HK = Hong Kong.

^c ^Six character reference codes used in IESLC. See Table two of [3] for associated reference(s).

**Table 6:** **Indirect estimates of mortality rates**^**a **^**by smoking habit – squamous lung cancer, males**

			**Never smoked**		**Former smoker**		**Current smoker**		**Ever smoker**	
**Region**	**Country**^**b**^	**Study**^**c**^	**Rate**	**Weight**	**Rate**	**Weight**	**Rate**	**Weight**	**Rate**	**Weight**
Canada		BAND	5.7	6.8					213.9	766.4
		JAIN	9.9	2.0	123.8	43.4	379.8	75.3	232.9	179.5
		SIEMIA	9.3	2.9					270.8	358.2
USA		BROWN2	24.1	184.9	214.7	1157.1	338.0	1108.7	268.1	2821.2
		BUFFLE	13.8	3.4					194.2	222.7
		BYERS1	26.9	22.2					223.4	514.8
		COMSTO	26.7	2.0	147.5	17.9	302.2	27.4	215.1	60.6
		DORGAN	14.3	4.0					269.9	526.3
		HAMMON	7.9	4.0					127.8	1033.6
		KHUDER	30.9	8.7	207.2	51.5	266.0	101.2	241.7	221.4
		OSANN	7.2	8.0	164.9	97.3	341.9	219.5	259.0	402.9
		STAYNE	51.0	22.2					177.0	164.2
		WYNDE2	10.9	2.9					215.5	804.0
		WYNDE3	17.6	3.0	148.5	33.3	425.9	203.8	321.4	482.9
		WYNDE4	8.3	7.6					106.6	1030.8
		WYNDE6	19.9	28.2	257.3	582.5	553.3	616.0	369.8	2394.8
SC America	Uruguay	DESTE2	18.3	3.6					240.9	124.4
	Cuba	JOLY	5.0	2.0					155.7	160.4
	Argentina	MATOS	14.3	3.0	48.7	14.2	131.4	32.9	87.3	57.7
	Argentina	PEZZOT	2.2	0.5					140.9	109.4
UK		ALDERS	24.8	1.8					364.3	290.6
		DOLL	12.1	2.9					157.5	943.0
Scandinavia	Sweden	DAMBER	15.1	12.7					178.3	143.0
	Norway	ENGELA	12.5	2.8	51.5	19.1	136.7	25.4	80.9	38.6
	Norway	KREYBE	1.7	2.3					21.3	8.9
	Sweden	NOU	6.1	2.0					167.0	63.5
W Europe	Italy	BARBON	13.8	5.9	122.1	50.7	242.0	189.5	198.6	314.1
	France	BENHAM	18.2	23.3	231.0	206.4	361.4	609.7	314.3	1174.6
	Germany	JAHN	8.7	3.0	187.5	180.9	225.6	124.5	201.3	462.2
	Austria	VUTUC	75.8	16.2					532.6	403.3
E Europe	Hungary	ABRAHA	2.8	0.5					260.5	187.3
	Poland	JEDRYC	18.9	5.9	131.0	42.3	413.5	225.6	291.1	346.4
	Hungary	ORMOS	6.5	2.0					66.3	29.5
	Poland	STASZE	1.1	0.5					62.1	108.3
Japan		MATSUD	2.6	1.0					102.4	214.8
		SOBUE	7.2	2.9	102.3	105.9	142.1	304.7	127.9	562.4
		WAKAI	18.4	2.0	115.6	26.1	181.5	108.3	159.7	188.8
China	HK	CHAN	18.3	1.9					278.9	65.5
	China	GAO	21.6	12.6					188.5	393.4
	HK	LAMWK2	43.6	4.5					300.2	76.2
	China	XU3	30.6	2.9					181.4	51.7
	China	ZHOU	71.8	41.0					225.2	73.9
Other	S Korea	CHOI	17.2	5.7					93.7	150.6

^a ^Mortality rates per 100,000 per year for age 70–74 years.

^b ^Not shown if same as region. HK = Hong Kong.

^c ^Six character reference codes used in IESLC. See Table two of [3] for associated reference(s).

**Table 7:** **Indirect estimates of mortality rates**^**a **^**by smoking habit – squamous lung cancer, females**

			**Never smoked**		**Former smoker**		**Current smoker**		**Ever smoker**	
**Region**	**Country**^**b**^	**Study**^**c**^	**Rate**	**Weight**	**Rate**	**Weight**	**Rate**	**Weight**	**Rate**	**Weight**
Canada		JAIN	2.7	5.9	21.4	18.6	77.3	49.2	49.7	78.7
USA		ANDERS	4.8	5.1					121.8	78.5
		BROWN2	5.3	115.8	103.0	225.7	110.5	304.6	107.2	592.2
		BUFFLE	2.5	3.0	31.1	17.3	32.5	45.2	32.0	93.9
		COMSTO	1.5	0.5	14.5	1.4	107.6	14.4	70.2	17.8
		DORGAN	5.5	24.3					61.2	206.3
		HAENSZ	10.7	51.8	19.5	2.3	33.0	46.2	32.3	51.9
		LOMBA2	5.6	15.3					23.8	124.6
		OSANN	3.3	12.1	44.3	26.4	106.1	114.1	84.6	155.9
		OSANN2	3.7	6.8	11.4	5.5	77.5	47.9	56.9	77.8
		WYNDE3	5.5	5.2					37.5	27.8
		WYNDE4	8.6	12.9					50.2	19.7
		WYNDE6	2.7	12.4	26.9	98.4	79.5	186.9	83.8	169.4
SC America	Cuba	JOLY	4.3	5.8					79.1	31.7
UK		ALDERS	9.8	7.1					59.7	91.9
		DOLL	16.0	17.4					38.5	33.7
Scandinavia	Norway	KREYBE	2.4	2.5					3.2	1.8
	Sweden	NOU	2.1	1.1					14.6	1.6
	Sweden	SVENSS	2.0	4.8	8.0	5.1	37.9	23.3	25.8	31.0
W Europe	Germany	BECHER	5.4	2.1					57.4	33.5
	France	BENHAM	10.6	36.4					60.0	17.8
	Greece	KATSOU	11.3	13.6	54.3	2.1	72.4	11.8	69.1	14.6
	Austria	VUTUC	11.8	35.0	130.0	24.0	216.4	38.8	179.4	60.0
E Europe	Hungary	ABRAHA	7.5	7.2					40.3	18.3
	Poland	STASZE	0.2	0.3					7.0	0.8
Japan		SOBUE	4.1	14.5	27.1	6.5	42.8	27.1	38.5	35.1
		WAKAI	2.9	3.1	27.7	0.8	80.0	11.3	71.5	12.8
China	HK	CHAN	24.2	16.2					155.7	22.5
	China	GAO	12.8	54.4					74.3	48.3
	K	KOO	41.7	26.3					172.9	31.5
	HK	LAMTH	20.7	23.2					168.0	16.8
	HK	LAMWK	11.0	6.8					115.5	14.5
	HK	LAMWK2	27.6	13.7					179.3	21.1
	China	WUWILL	15.5	59.3					65.0	134.9
	China	XU3	9.9	2.1					168.1	9.9
	China	ZHOU	23.6	37.9					90.1	7.0
Other	S Korea	CHOI	4.3	10.1					29.6	8.2
	Singapore	SEOW	9.9	8.5					172.7	8.1

^a ^Mortality rates per 100,000 per year for age 70–74 years.

^b ^Not shown if same as region. HK = Hong Kong.

^c ^Six character reference codes used in IESLC. See Table two of [3] for associated reference(s).

**Table 8:** **Indirect estimates of mortality rates**^**a **^**by smoking habit – adeno lung cancer, males**

			**Never smoked**		**Former smoker**		**Current smoker**		**Ever smoker**	
**Region**	**Country**^**b**^	**Study**^**c**^	**Rate**	**Weight**	**Rate**	**Weight**	**Rate**	**Weight**	**Rate**	**Weight**
Canada		BAND	40.7	42.8					166.8	626.5
		JAIN	19.7	3.9	79.0	28.5	213.0	48.5	136.1	98.1
		SIEMIA	15.5	4.8					123.2	162.4
USA		BROWN2	20.4	153.9	152.7	791.0	187.8	628.3	167.6	1700.3
		BUFFLE	25.8	5.3					115.9	115.2
		BYERS1	8.6	7.0					35.1	50.3
		COMSTO	26.7	2.0	112.8	13.5	335.8	30.5	210.2	58.7
		DORGAN	17.7	4.8					85.1	126.1
		HAMMON	3.9	2.0					13.0	31.8
		KHUDER	24.1	6.8	192.8	52.5	197.6	65.1	195.4	165.0
		OSANN	12.6	14.0	160.1	94.7	300.4	195.5	234.7	360.2
		STAYNE	16.2	7.0					58.5	46.2
		WYNDE2	18.2	4.8					30.4	47.2
		WYNDE3	35.2	5.8	49.5	11.7	139.5	59.1	105.6	83.7
		WYNDE4	4.1	3.9					6.2	35.9
		WYNDE6	39.8	54.9	153.8	371.2	349.7	458.4	228.8	1297.1
SC America	Uruguay	DESTE2	26.2	4.0					112.5	78.9
	Cuba	JOLY	12.5	4.9					55.2	65.8
	Argentina	MATOS	24.5	5.0	117.6	33.8	187.6	45.6	150.3	113.3
	Argentina	PEZZOT	13.7	3.0					99.9	71.6
UK		ALDERS	14.2	1.8					101.2	43.1
		DOLL	7.1	1.9					6.4	31.9
Scandinavia	Sweden	DAMBER	17.6	11.7					42.3	41.6
	Norway	ENGELA	14.1	4.4	15.5	9.3	99.8	13.0	32.9	16.8
	Norway	KREYBE	1.7	2.3					4.3	7.6
	Sweden	NOU	12.3	3.5					54.7	16.4
W Europe	Italy	BARBON	16.1	6.9	88.4	38.1	129.9	105.0	114.9	167.4
	France	BENHAM	8.4	7.7	20.4	18.1	35.0	60.3	30.1	96.4
	Germany	JAHN	23.3	7.7	123.7	122.5	109.1	67.3	117.0	237.2
	Austria	VUTUC	8.9	2.0					58.3	39.5
E Europe	Hungary	ABRAHA	45.3	8.1					108.6	65.7
	Poland	JEDRYC	22.0	6.7	56.3	19.0	110.2	54.6	85.5	72.2
	Hungary	ORMOS	1.3	0.4					8.8	3.6
	Poland	STASZE	1.0	0.5					8.9	18.8
Japan		MATSUD	1.3	0.5					22.4	26.2
		SOBUE	64.7	23.1	98.9	102.8	130.3	279.1	119.1	512.0
		WAKAI	73.8	7.4	98.5	22.4	158.3	91.4	138.5	150.4
China	HK	CHAN	4.5	0.5					136.0	40.8
	China	GAO	69.7	38.0					108.1	203.5
	HK	LAMWK2	130.7	11.2					121.0	40.6
	China	XU3	30.6	2.9					134.9	35.5
	China	ZHOU	65.8	39.4					86.0	54.8
Other	S Korea	CHOI	20.1	6.6					26.9	45.2

^a ^Mortality rates per 100,000 per year for age 70–74 years.

^b ^Not shown if same as region. HK = Hong Kong.

^c ^Six character reference codes used in IESLC. See Table two of [3] for associated reference(s).

**Table 9:** **Indirect estimates of mortality rates**^**a **^**by smoking habit – adeno lung cancer, females**

			**Never smoked**		**Former smoker**		**Current smoker**		**Ever smoker**	
**Region**	**Country**^**b**^	**Study**^**c**^	**Rate**	**Weight**	**Rate**	**Weight**	**Rate**	**Weight**	**Rate**	**Weight**
Canada		JAIN	10.6	22.6	16.6	14.9	65.9	44.5	41.5	68.4
USA		ANDERS	31.4	36.8					191.4	143.6
		BROWN2	18.9	413.3	132.1	336.8	132.1	336.8	130.7	748.8
		BUFFLE	9.8	20.0	32.7	17.5	46.7	61.6	39.6	130.4
		COMSTO	25.7	8.5	63.4	5.6	163.5	21.4	123.2	33.3
		DORGAN	13.2	48.4					51.4	217.4
		HAENSZ	9.0	42.4	12.8	1.7	9.8	15.3	10.0	17.4
		LOMBA2	20.2	57.9					10.7	47.2
		OSANN	12.9	48.7	76.4	42.6	157.6	159.9	129.3	235.9
		OSANN2	11.1	19.0	19.8	6.3	38.6	25.2	33.0	39.6
		WYNDE3	16.6	17.3					31.5	23.0
		WYNDE4	11.2	18.4					6.7	2.1
		WYNDE6	13.0	62.4	65.6	126.3	156.5	265.1	178.5	250.4
SC America	Cuba	JOLY	17.8	21.7					54.4	24.4
UK		ALDERS	8.2	8.6					29.5	24.4
		DOLL	3.3	4.5					6.4	6.7
Scandinavia	Norway	KREYBE	21.7	9.6					16.1	5.9
	Sweden	NOU	29.8	15.6					26.3	6.8
	Sweden	SVENSS	8.8	18.5	16.0	9.0	34.3	22.1	26.9	31.8
W Europe	Germany	BECHER	2.9	1.0					31.7	17.4
	France	BENHAM	5.8	18.2					14.0	3.9
	Greece	KATSOU	24.3	28.2	40.7	1.8	45.2	9.1	44.4	11.3
	Austria	VUTUC	19.6	52.8	31.2	9.5	29.2	12.0	30.1	21.1
E Europe	Hungary	ABRAHA	17.2	17.2					45.1	20.7
	Poland	STASZE	8.6	13.9					9.4	1.0
Japan		SOBUE	39.7	204.0	77.5	16.1	56.1	34.8	62.0	55.6
		WAKAI	44.0	81.8	110.9	2.3	48.0	7.5	58.1	10.8
China	HK	CHAN	50.9	29.4					99.1	17.4
	China	GAO	64.3	305.7					69.8	46.1
	HK	KOO	60.0	35.0					96.4	22.3
	HK	LAMTH	100.4	73.4					187.6	31.3
	HK	LAMWK	94.0	49.2					198.0	20.5
	HK	LAMWK2	75.5	32.8					133.2	17.4
	China	WUWILL	45.7	184.2					62.8	124.3
	China	XU3	34.5	7.9					44.8	3.5
	China	ZHOU	54.0	77.0					77.2	6.8
Other	S Korea	CHOI	20.9	50.9					13.5	4.3
	Singapore	SEOW	66.1	30.8					156.2	7.8

^a ^Mortality rates per 100,000 per year for age 70-74 years.

^b ^Not shown if same as region. HK = Hong Kong.

^c ^Six character reference codes used in IESLC. See Table two of [3] for associated reference(s).

### Meta-analyses

Results of the meta-analyses, overall and by sex, region and year of study, are shown in Table 10 (never smokers), Table 11 (ever smokers), Table 12 (current smokers) and Table 13 (former smokers). In the text below, all rates mentioned are per 100,000 per year. Estimates given are random-effects and usually presented to 3 significant figures together with the 95% confidence interval (CI) and the number of individual estimates they were based on, (e.g. 258, 237–278, n = 220).

**Table 10:** Meta-analyses of indirect estimates of lung cancer mortality rates in never smokers

**Sex**	**Region**	**Period**	**Statistic**^**a**^	**All lung cancer**^**b**^	**Squamous**^**c**^	**Adeno**^**d**^
All	All	All	n	220	81	81
			Rate	45.8 (41.7–50.4)	10.5 (8.6–12.8)	21.2 (17.9–25.1)
			H,P_H_	23.53, <0.001	9.18, <0.001	16.21, <0.001
Male			n	129	43	43
			Rate	56.3 (49.8–63.7)	15.5 (12.2–19.8)	20.2 (15.8–25.8)
Female			n	91	38	38
			Rate	36.0 (31.6–41.0)	7.6 (6.0–9.7)	22.1 (17.5–28.0)
Between^e^			P_B_	<0.001	<0.001	NS
	Canada		n	10	4	4
			Rate	34.5 (24.3–48.9)	5.4 (2.9–9.7)	19.4 (8.5–44.4)
	USA		n	54	25	25
			Rate	37.6 (32.6–43.3)	9.3 (6.5–13.4)	16.1 (13.5–19.1)
	SC America^f^		n	9	5	5
			Rate	40.2 (28.4–56.7)	7.9 (3.8–16.2)	18.0 (13.2–24.7)
	UK		n	26	4	4
			Rate	61.5 (46.8–80.8)	14.2 (9.9–20.4)	6.7 (3.8–11.6)
	Scandinavia^g^		n	20	7	7
			Rate	29.6 (21.9–40.0)	4.4 (1.9–10.2)	13.2 (7.8–22.6)
	W Europe^h^		n	31	8	8
			Rate	38.2 (29.3–49.8)	14.9 (8.9–24.9)	13.1 (8.3–20.7)
	E Europe^i^		n	11	6	6
			Rate	32.3 (22.3–46.8)	6.6 (2.8–15.5)	14.7 (7.3–29.8)
	Japan		n	14	5	5
			Rate	42.5 (34.5–52.4)	5.0 (2.8–8.7)	47.2 (35.3–63.0)
	China^j^		n	38	14	14
			Rate	99.1 (90.2–108.8)	23.7 (16.8–33.4)	64.8 (54.6–76.9)
	Other Asia^k^		n	7	3	3
			Rate	23.5 (9.9–55.8)	8.7 (3.9–19.2)	31.0 (13.1–73.4)
	Between^e^		P_B_	<0.001	<0.05	<0.001
		1930–60	n	36	11	11
			Rate	24.1 (20.2–28.9)	7.6 (5.1–11.2)	6.9 (4.6–10.4)
		1961–70	n	26	8	8
			Rate	41.2 (31.2–54.4)	12.6 (6.0–26.7)	17.0 (12.6–22.9)
		1971–80	n	46	18	18
			Rate	50.2 (39.8–63.2)	12.7 (8.6–18.9)	18.1 (11.8–27.8)
		1981–90	n	81	40	40
			Rate	59.5 (52.1–67.9)	10.2 (7.6–13.6)	29.0 (23.4–35.8)
		1991–99	n	31	4	4
			Rate	44.4 (33.9–58.0)	11.6 (7.3–18.4)	33.9 (17.6–65.3)
		Between^e^	P_B_	<0.001	NS	<0.01

^a ^n = number of estimates combined, Rate = random-effects meta-analysis lung cancer mortality rate per 100,000 per year for age 70-74 years (95% CI), H = heterogeneity per degree of freedom, P_H_ = probability value for heterogeneity expressed as p < 0.001, p < 0.01, p < 0.05, p < 0.1 or NS (p ≥ 0.1), P_B_ = probability value for heterogeneity between levels (see Methods) similarly expressed.

^b ^All or nearest available, must include at least squamous cell carcinoma and adenocarcinoma.

^c ^Squamous cell carcinoma or nearest available, but not including adenocarcinoma.

^d ^Adenocarcinoma or nearest available, but not including squamous cell carcinoma.

^e ^Heterogeneity between levels of factor considered.

^f ^Including Argentina, Brazil, Cuba, Uruguay.

^g ^Including Denmark, Finland, Iceland, Norway, Sweden.

^h ^Including Austria, Belgium, France, Germany, Greece, Italy, Netherlands, Spain, Switzerland.

^i ^Including Czechoslovakia, Hungary, Poland.

^j ^Including China, Hong Kong.

^k ^Including Singapore, South Korea, Thailand.

**Table 11:** Meta-analyses of indirect estimates of lung cancer mortality rates in ever smokers

**Sex**	**Region**	**Period**	**Statistic**^**a**^	**All lung cancer**^**b**^	**Squamous**^**c**^	**Adeno**^**d**^
All	All	All	n	220	81	81
			Rate	258.3 (239.6–278.3)	117.0 (102.7–133.3)	58.5 (50.1–68.2)
			H,P_H_	244.49, <0.001	83.73, <0.001	52.65, <0.001
Male			n	129	43	43
			Rate	365.9 (334.4–400.4)	185.9 (163.1–211.9)	67.1 (55.3–81.4)
Female			n	91	38	38
			Rate	148.2 (130.5–168.4)	65.2 (54.3–78.4)	49.1 (38.2–63.1)
Between^e^			P_B_	<0.001	<0.001	<0.01
	Canada		n	10	4	4
			Rate	223.4 (153.2–325.7)	162.8 (102.0–259.8)	104.7 (64.3–170.5)
	USA		n	54	25	25
			Rate	286.7 (246.0–334.2)	115.9 (90.9–147.6)	63.4 (48.3–83.2)
	SC America^f^		n	9	5	5
			Rate	320.1 (253.8–403.7)	131.9 (92.6–188.0)	88.7 (60.2–130.6)
	UK		n	26	4	4
			Rate	352.3 (294.5–421.6)	108.7 (52.4–225.7)	18.9 (4.5–79.8)
	Scandinavia^g^		n	20	7	7
			Rate	199.9 (149.0–268.2)	43.8 (21.2–90.6)	24.6 (14.9–40.4)
	W Europe^h^		n	31	8	8
			Rate	245.0 (203.3–295.3)	160.8 (112.3–230.4)	46.9 (28.6–76.7)
	E Europe^i^		n	11	6	6
			Rate	255.4 (178.3–365.9)	89.4 (44.4–180.2)	30.2 (13.2–68.9)
	Japan		n	14	5	5
			Rate	151.0 (115.5–197.6)	94.5 (69.1–129.3)	69.2 (43.3–110.6)
	China^j^		n	38	14	14
			Rate	315.6 (291.6–341.5)	156.5 (117.5–208.4)	106.5 (87.4–129.7)
	Other Asia^k^		n	7	3	3
			Rate	67.6 (26.1–174.8)	79.5 (36.7–172.2)	39.0 (11.2–135.7)
	Between^e^		P_B_	<0.001	NS	<0.05
		1930–60	n	36	11	11
			Rate	120.7 (103.7–140.6)	59.6 (45.8–77.6)	8.2 (6.5–10.4)
		1961–70	n	26	8	8
			Rate	203.4 (161.2–256.8)	110.7 (68.4–179.1)	33.8 (19.4–58.7)
		1971–80	n	46	18	18
			Rate	285.5 (246.1–331.2)	143.5 (101.9–202.2)	55.7 (42.1–73.6)
		1981–90	n	81	40	40
			Rate	340.6 (312.8–370.9)	125.3 (105.4–148.9)	97.9 (84.7–113.2)
		1991–99	n	31	4	4
			Rate	326.5 (283.8–375.5)	165.6 (111.5–246.1)	126.7 (108.4–148.0)
		Between^e^	P_B_	<0.001	<0.01	<0.001

^a ^n = number of estimates combined, Rate = random-effects meta-analysis lung cancer mortality rate per 100,000 per year for age 70–74 years (95% CI), H = heterogeneity per degree of freedom, P_H_ = probability value for heterogeneity expressed as p < 0.001, p < 0.01, p < 0.05, p < 0.1 or NS (p ≥ 0.1), P_B_ = probability value for heterogeneity between levels (see Methods) similarly expressed.

^b ^All or nearest available, must include at least squamous cell carcinoma and adenocarcinoma.

^c ^Squamous cell carcinoma or nearest available, but not including adenocarcinoma.

^d ^Adenocarcinoma or nearest available, but not including squamous cell carcinoma.

^e ^Heterogeneity between levels of factor considered.

^f ^Including Argentina, Brazil, Cuba, Uruguay.

^g ^Including Denmark, Finland, Iceland, Norway, Sweden.

^h ^Including Austria, Belgium, France, Germany, Greece, Italy, Netherlands, Spain, Switzerland.

^i ^Including Czechoslovakia, Hungary, Poland.

^j ^Including China, Hong Kong.

^k ^Including Singapore, South Korea, Thailand.

**Table 12:** Meta-analyses of indirect estimates of lung cancer mortality rates in current smokers

**Sex**	**Region**	**Period**	**Statistic**^**a**^	**All lung cancer**^**b**^	**Squamous**^**c**^	**Adeno**^**d**^
All	All	All	n	116	28	28
			Rate	369.8 (328.0–416.9)	148.8 (114.7–193.1)	102.1 (81.3–128.1)
			H,P_H_	160.43, <0.001	78.07, <0.001	38.44, <0.001
Male			n	70	15	15
			Rate	546.6 (472.3–632.4)	275.3 (224.1–338.3)	157.5 (120.5–205.7)
Female			n	46	13	13
			Rate	196.9 (159.1–243.7)	71.9 (54.9–94.2)	59.7 (41.8–85.2)
Between^e^			P_B_	<0.01	<0.001	<0.05
	Canada		n	5	2	2
			Rate	314.1 (161.0–612.7)	171.8 (36.1–817.1)	118.5 (37.5–374.4)
	USA		n	40	13	13
			Rate	476.7 (390.8–581.5)	150.4 (97.1–233.0)	129.0 (94.6–175.7)
	SC America^f^		n	8	1	1
			Rate	381.5 (290.7–500.5)	131.4 (93.4–185.0)	187.6 (140.3–250.8)
	UK		n	16	0	0
			Rate	406.6 (303.3–545.1)		
	Scandinavia^g^		n	12	2	2
			Rate	381.7 (257.1–566.9)	72.1 (20.5–253.2)	57.7 (20.3–164.0)
	W Europe^h^		n	17	5	5
			Rate	342.5 (244.0–480.7)	217.0 (156.6–300.8)	59.7 (31.8–111.9)
	E Europe^i^		n	2	1	1
			Rate	425.3 (88.8–2036.7)	413.5 (363.0–471.2)	110.2 (84.5–143.6)
	Japan		n	9	4	4
			Rate	187.0 (133.1–262.9)	101.1 (62.5–163.6)	94.1 (61.3–144.6)
	China^j^		n	3	0	0
			Rate	285.1 (198.3–410.0)		
	Other Asia^k^		n	4	0	0
			Rate	130.3 (67.3–252.3)		
	Between^e^		P_B_	<0.05	NS	NS
		1930–60	n	10	1	1
			Rate	140.6 (112.3–176.0)	33.0 (24.8–44.1)	9.8 (5.9–16.1)
		1961–70	n	17	2	2
			Rate	331.2 (247.4–443.4)	244.9 (80.4–745.8)	129.1 (97.9–170.2)
		1971–80	n	24	4	4
			Rate	382.4 (289.5–505.1)	118.9 (36.9–383.4)	38.9 (32.6–46.5)
		1981–90	n	49	19	19
			Rate	457.4 (393.5–531.6)	157.5 (115.1–215.7)	135.3 (108.9–168.1)
		1991–99	n	16	2	2
			Rate	400.8 (293.1–548.1)	175.8 (103.7–298.1)	142.1 (83.6–241.7)
		Between^e^	P_B_	<0.001	NS	<0.01

^a ^n = number of estimates combined, Rate = random-effects meta-analysis lung cancer mortality rate per 100,000 per year for age 70–74 years (95% CI), H = heterogeneity per degree of freedom, P_H_ = probability value for heterogeneity expressed as p < 0.001, p < 0.01, p < 0.05, p < 0.1 or NS (p ≥ 0.1), P_B_ = probability value for heterogeneity between levels (see Methods) similarly expressed.

^b ^All or nearest available, must include at least squamous cell carcinoma and adenocarcinoma.

^c ^Squamous cell carcinoma or nearest available, but not including adenocarcinoma.

^d ^Adenocarcinoma or nearest available, but not including squamous cell carcinoma.

^e ^Heterogeneity between levels of factor considered.

^f ^Including Argentina, Brazil, Cuba, Uruguay.

^g ^Including Denmark, Finland, Iceland, Norway, Sweden.

^h ^Including Austria, Belgium, France, Germany, Greece, Italy, Netherlands, Spain.

^i ^Including Czechoslovakia, Poland.

^j ^Including China, Hong Kong.

^k ^Including Singapore, South Korea.

**Table 13:** Meta-analyses of indirect estimates of lung cancer mortality rates in former smokers

**Sex**	**Region**	**Period**	**Statistic**^**a**^	**All lung cancer**^**b**^	**Squamous**^**c**^	**Adeno**^**d**^
All	All	All	n	116	28	28
			Rate	197.7 (177.2–220.6)	78.6 (61.0–101.3)	68.0 (55.7–83.0)
			H,P_H_	35.58, <0.001	36.48, <0.001	16.78, <0.001
Male			n	70	15	15
			Rate	277.1 (246.4–311.6)	144.3 (121.2–171.8)	91.4 (74.1–112.8)
Female			n	46	13	13
			Rate	105.1 (87.3–126.6)	31.2 (18.6–52.4)	43.4 (28.4–66.3)
Between^e^			P_B_	<0.001	<0.001	<0.05
	Canada		n	5	2	2
			Rate	129.5 (64.9–258.5)	52.0 (9.3–289.8)	36.6 (7.9–169.0)
	USA		n	40	13	13
			Rate	230.8 (193.3–275.5)	79.7 (53.9–118.0)	90.9 (72.4–114.2)
	SC America^f^		n	8	1	1
			Rate	216.5 (167.0–280.7)	48.7 (29.0–82.1)	117.6 (84.0–164.8)
	UK		n	16	0	0
			Rate	220.3 (159.1–305.1)		
	Scandinavia^g^		n	12	2	2
			Rate	109.3 (71.8–166.4)	21.0 (3.4–130.6)	15.7 (10.0–24.9)
	W Europe^h^		n	17	5	5
			Rate	217.1 (165.4–284.9)	160.7 (121.4–212.7)	51.3 (24.6–107.2)
	E Europe^i^		n	2	1	1
			Rate	196.2 (69.4–554.5)	131.0 (96.9–177.1)	56.3 (35.9–88.2)
	Japan		n	9	4	4
			Rate	178.5 (140.4–226.9)	74.0 (43.2–126.6)	96.4 (81.8–113.5)
	China^j^		n	3	0	0
			Rate	334.4 (276.2–405.0)		
	Other Asia^k^		n	4	0	0
			Rate	72.7 (52.5–100.7)		
	Between^e^		P_B_	NS	NS	<0.1
		1930–60	n	10	1	1
			Rate	105.0 (72.3–152.4)	19.5 (5.3–71.3)	12.8 (2.8–58.5)
		1961–70	n	17	2	2
			Rate	152.0 (105.8–218.3)	88.3 (31.3–249.5)	28.0 (9.0–87.1)
		1971–80	n	24	4	4
			Rate	226.1 (176.4–289.9)	60.1 (18.9–190.6)	25.8 (19.6–33.9)
		1981–90	n	49	19	19
			Rate	229.7 (198.5–265.8)	80.9 (58.7–111.6)	87.0 (71.2–106.2)
		1991–99	n	16	2	2
			Rate	193.4 (148.9–251.2)	97.9 (26.2–366.3)	122.3 (104.6–143.1)
		Between^e^	P_B_	<0.001	NS	<0.05

^a ^n = number of estimates combined, Rate = random-effects meta-analysis lung cancer mortality rate per 100,000 per year for age 70–74 years (95% CI), H = heterogeneity per degree of freedom, P_H_ = probability value for heterogeneity expressed as p < 0.001, p < 0.01, p < 0.05, p < 0.1 or NS (p ≥ 0.1), P_B_ = probability value for heterogeneity between levels (see Methods) similarly expressed.

^b ^All or nearest available, must include at least squamous cell carcinoma and adenocarcinoma.

^c ^Squamous cell carcinoma or nearest available, but not including adenocarcinoma.

^d ^Adenocarcinoma or nearest available, but not including squamous cell carcinoma.

^e ^Heterogeneity between levels of factor considered.

^f ^Including Argentina, Brazil, Cuba, Uruguay.

^g ^Including Denmark, Finland, Iceland, Norway, Sweden.

^h ^Including Austria, Belgium, France, Germany, Greece, Italy, Netherlands, Spain.

^i ^Including Czechoslovakia, Poland.

^j ^Including China, Hong Kong.

^k ^Including Singapore, South Korea.

### Never smokers

There are 220 estimates of all lung cancer risk in never smokers, yielding an overall random-effects estimate of 45.8 (41.7–50.4). There is marked heterogeneity (p < 0.001), with estimates varying from a minimum of 1.7 (SINARA, Thailand, females) to a maximum of 655 (GREGOR, UK, males). Rates are higher (p < 0.001) in males (56.3, 49.8–63.7, n = 129) than in females (36.0, 31.6–41.0, n = 91). There is also significant (p < 0.001) variation by region, with rates clearly higher in China (99.1, 90.2–109, n = 38) than in the other nine regions studied, where estimates vary from 23.5 to 61.5. The difference between the sexes is evident in each region, except for other Asia, where there are few estimates (data not shown). Even for China, where rates in females are particularly high (89.8, 82.5–97.8, n = 20), rates are still higher in males (119, 104–136, n = 18). While there is a significant (p < 0.001) evidence of variation by period of study, the trend it not simple, with rates starting low in 1930–1960, increasing to 1981–1990 and then falling.

There are 81 estimates for squamous in never smokers, with the overall rate estimate 10.5 (8.6–12.8), 23% of the total lung cancer risk. There is a clearly (p < 0.001) higher risk for males (15.5, 12.2–19.8, n = 43) than for females (7.6, 6.0–9.7, n = 38). The variation by region is less clear (p < 0.05), though rates were again highest for China not only overall (23.7, 16.8–33.4, n = 14), but also separately in males (35.7, 18.3–69.6, n = 5) and females (20.1, 15.0–26.8, n = 9). There is no significant variation by period (p ≥ 0.1) with rates quite similar between 1961–70 and 1991–98.

The 81 estimates for adeno in never smokers gave an estimate of 21.2 (17.9–25.1), higher than that for squamous, forming 46% of the total lung cancer risk. Here there is no evidence of a difference between the sexes (p ≥ 0.1) with rates 20.2 (15.8–25.8, n = 43) for males and 22.1 (17.5–28.0, n = 38) for females. Rates clearly vary by region, being higher in China (64.8, 54.6–76.9, n = 14), Japan (47.2, 35.3–63.0, n = 5) and other Asian countries (31.0, 13.1–73.4) than in other regions, where rate estimates vary from 6.7 to 19.4. Rates in China and in Japan are quite similar in males and females (data not shown). There is also evidence of variation by period (p < 0.01), with rates rising steadily from 6.9 (4.6–10.4, n = 11) for 1930–60, to 33.9 (17.6–65.3, n = 4) for 1991–98.

### Ever smokers

The estimated rates shown in Table 11 for ever smokers are substantially higher than those for never smokers in Table 10. Thus the all lung cancer rate for ever smokers of 258 (240–278, n = 220) is 5.6 times the rate for never smokers, while those of 117 (103–133, n = 81) for squamous and 58.5 (50.1–68.2, n = 81) for adeno are, respectively 11.1 times and 2.8 times the corresponding rates for never smokers. Whereas, in never smokers, rates are about twice as high for adeno than for squamous, the reverse is true for ever smokers, with rates for squamous double those for adeno.

The difference between the sexes is clearer for ever smokers than for never smokers. For ever smokers, rates in males are 147% higher than in females for all lung cancer (p < 0.001), 185% higher for squamous (p < 0.001) and 37% higher for adenocarcinoma (p < 0.01). For never smokers the corresponding excesses in males compared to females are 56% for all lung cancer and 104% for squamous, with no excess seen for adenocarcinoma.

There is clear variation (p < 0.001) in ever smoker all lung cancer rates by region. However, while rates are, as for never smokers, high in China (316, 292–342, n = 38), they are similar in the UK (352, 295–422, n = 26) and almost as high in South and Central America (320, 254–404, n = 9) and in the USA (287, 246–334, n = 341). Variation by region in ever smoker rates is not significant (p ≥ 0.1) for squamous, but is significant (p < 0.05) for adeno. Rates in China remain relatively high for both lung cancer types, though as for all lung cancer, some regions have similar rates.

There is a tendency for rates to rise over time, particularly for all lung cancer (p < 0.001) and adeno (p < 0.001) and evident to some extent for squamous (p < 0.01). The rise is particularly striking for adeno, where rates are 8.2, 33.8, 55.7, 97.9 and 127 for the five successive periods studied.

### Trends in rates for never and ever smokers by region

**Figure 1:**
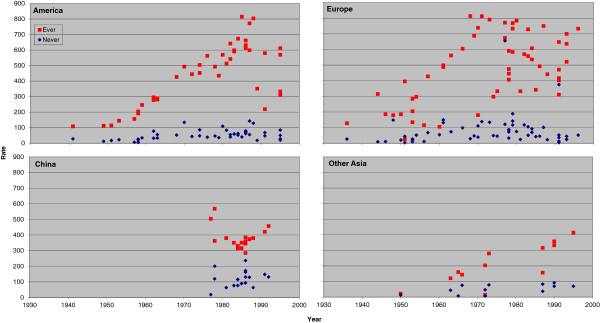
**Scatter plot of lung cancer rates in males for never and ever smokers. **Table 4 presents indirect estimates of mortality rates (per 100,000 per year) by smoking habit for all lung cancer in males. The individual study estimates for never smokers (blue diamonds) and ever smokers (red squares) are plotted against the midpoint year of the study, with separate plots shown for America (Canada, US and South/Central America), Europe (UK, Scandinavia, West Europe and East Europe), China (including Hong Kong), and Other Asia (Japan, South Korea, Singapore and Thailand).

**Figure 2:**
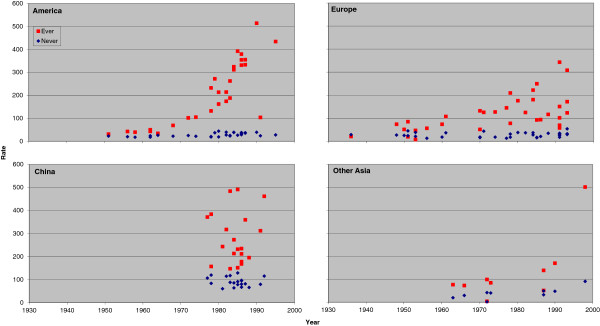
**Scatter plot of lung cancer rates in females for never and ever smokers. **Figure 2 is laid out as Figure 1 except that the scale of the y-axis extends up to 600 rather than up to 900. The individual study estimates are as given in Table 5.

Figure 1 (males) and Figure 2 (females) plot the individual rate estimates for all lung cancer by study midpoint year separately for the four major regions: America, Europe, China and other Asian countries. Estimates for ever and never smokers are distinguished by colour. A number of features of the results are clear, some already referred to in the preceding sections. These include the higher rates in ever smokers than never smokers; the higher rates in never smokers in China than elsewhere; the clear tendency for ever smoker rates to rise with time in America and Europe, any corresponding time trend in China not being evident perhaps due to the time range studied there being much narrower; and the lack of any very clear time trend in never smokers, except that rates before 1960 are lower.

**Figure 3:**
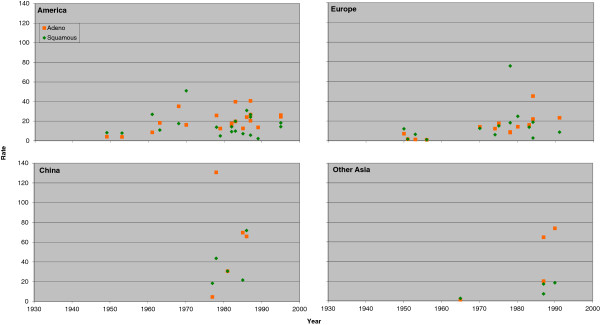
**Scatter plot of lung cancer rates by histological type in males for never smokers.** Table 6 (squamous) and Table 8 (adeno) present indirect estimates of mortality rates (per 100,000 per year) in male never smokers by histological type. The individual study estimates for squamous (green diamonds) and adeno (orange squares) are plotted against the midpoint year of the study, with separate plots shown for America (Canada, US and South/Central America), Europe (UK, Scandinavia, West Europe and East Europe), China (including Hong Kong), and Other Asia (Japan and South Korea, Singapore and Thailand).

**Figure 4:**
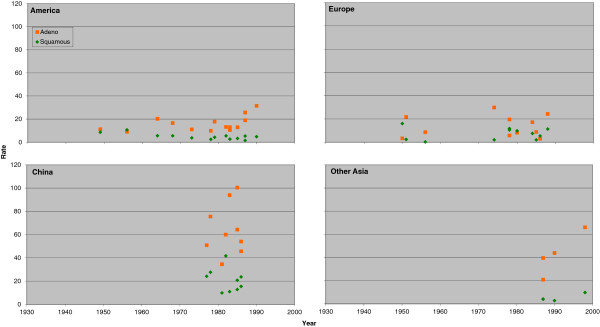
**Scatter plot of lung cancer rates by histological type in females for never smokers.** Figure 4 is laid out as Figure 3 except that the scale of the y-axis extends up to 120 rather than up to 140. The individual study estimates are as given in Tables 7 and 9.

**Figure 5:**
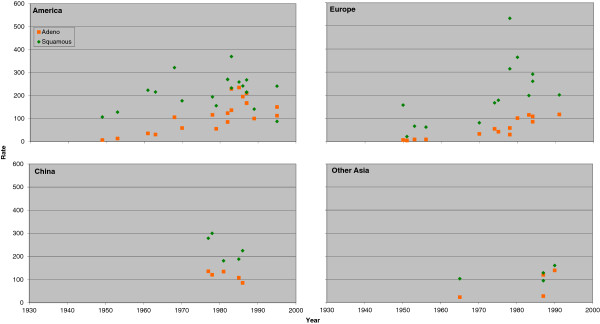
**Scatter plot of lung cancer rates by histological type in males for ever smokers.** Table 6 (squamous) and Table 8 (adeno) presents indirect estimates of mortality rates (per 100,000 per year) in male ever smokers by histological type. The individual study estimates for squamous (green diamonds) and adeno (orange squares) are plotted against the midpoint year of the study, with separate plots shown for America (Canada, US and South/Central America), Europe (UK, Scandinavia, West Europe and East Europe), China (including Hong Kong), and Other Asia (Japan, South Korea, Singapore and Thailand).

**Figure 6:**
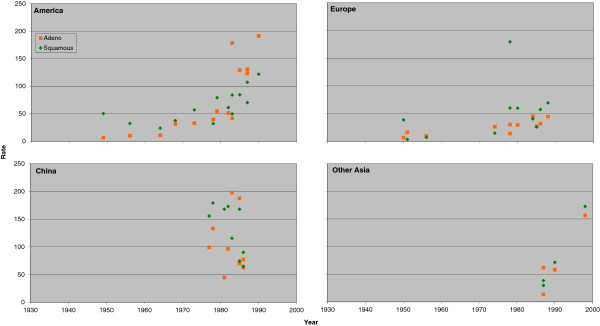
**Scatter plot of lung cancer rates by histological type in females for ever smokers.** Figure 4 is laid out as Figure 3 except that the scale of the y-axis extends up to 120 rather than up to 140. The individual study estimates are as given in Tables 7 and 9.

Figure 3 (males) and Figure 4 (females) plot the individual rate estimates for never smokers by study midpoint year for the same four regions, with estimates for squamous and adeno distinguished by colour. Figure 5 (males) and Figure 6 (females) similarly plot results for ever smokers. In never smokers, rates are generally higher for adeno than squamous, with the reverse being true for ever smokers. While never smokers adeno rates are particularly high in China, (most clearly seen for females), never smoker squamous rates are also higher in China than elsewhere. For both never and ever smokers, evidence of an increasing time trend is stronger for adeno than squamous.

### Current smokers

The estimated rates shown in Table 12 for current smokers are higher than the corresponding rates for ever smokers in Table 11. Thus the rates are 370 (328–417, n = 116) for all lung cancer, 149 (115–193, n = 28) for squamous and 102 (81.3–128, n = 28) for adeno, which are, respectively, 43%, 27% and 75% higher than the corresponding rates for ever smokers. Rates in current smokers are clearly higher in males than in females for all lung cancer (p < 0.01), squamous (p < 0.001) and adeno (p < 0.05): For squamous the rate in males of 275 (224–338, n = 15) is almost 4 times that for females of 71.9 (54.9–94.2, n = 13).

For all lung cancer, there is significant (p < 0.05) variation by region. Rates are highest in the USA (477, 391–582, n = 40) and exceed 300 in all European and American regions, but are lower in Asia. Since there are only 28 estimates for current smokers by lung cancer type, with 13 from the USA, there are insufficient data to see a clear pattern by region. No significant relationship was noted (p ≥ 0.1) for either squamous or adeno.

For all lung cancer, there was significant (p < 0.001) variation by period, with the rates of 141 (113–176, n = 10) for 1930–60, rising to a high of 457 (394–532, n = 49) for 1981–90. Clear patterns by period are not evident by lung cancer type, partly because 19 of the 28 estimates are for the period 1981–90. A significant relationship was not seen for squamous (p ≥ 0.1), but was seen for adeno (p < 0.01), this being due to lower rates (<50) for 1930–60 and 1971–80, and higher rates (>100) for other periods.

### Former smokers

The estimated rates shown in Table 13 for former smokers are lower than the corresponding rates for current smokers in Table 12. Thus the rates are 198 (177–221, n = 116) for all lung cancer, 78.6 (61.0–101, n = 28) for squamous and 68.0 (55.7–83.0, n = 28) for adeno, which are, respectively, 53%, 53% and 67% of the corresponding estimates for current smokers. As for current smokers, rates in former smokers were clearly higher for males than females for all lung cancer (p < 0.001), squamous (p < 0.001) and adeno (p < 0.05), with the excess particularly marked for squamous, where the rate was 144 (121–172, n = 15) in males and 31.2 (18.6–52.4, n = 13) in females.

There was no significant variation (p ≥ 0.1) by region in all lung cancer rates for former smokers. Limited data for regions other than the USA made variations by lung cancer type difficult to assess.

There was evidence of variation by period, due mainly to a tendency for rates to increase with time, for all lung cancer (p < 0.001) and adeno (p < 0.05), but not squamous (p ≥ 0.1).

### Meta-regressions

The preceding sections report rates, for a given smoking status and endpoint, overall and by sex, region and period. Although limited results are given jointly by sex and region (China/not China) for never smokers, the tables and text describing them predominantly concern variation by sex, region and period considered independently. There are, however, considerable correlations between the factors. For example, based on the 220 estimates for ever or never smoking for all lung cancer, the 59 estimates for Asia include a higher proportion of estimates for females (49%) and for 1981–1998 (68%) than is the case for the 161 estimates for other regions, where the proportions are 38% for females and 45% for 1981–1998.

Table 14 presents the results of inverse-variance weighted regression analyses for never smokers. There is clear evidence of variation by continent, highly significant (p < 0.001) for five of the six analyses, and less significant (p < 0.05) for squamous in males. Rates are similar in Europe and America, and clearly lower than in China. Rates in Asia (not China) are also consistently lower than in China.

**Table 14:** Inverse-variance weighted regression analyses – never smokers

	**Males**			**Females**		
	**All**	**Squamous**	**Adeno**	**All**	**Squamous**	**Adeno**
Overall deviance (d.f.)	1525 (128)	196 (42)	254 (42)	2766 (90)	319 (37)	1040 (37)
Drop in deviance and p value^a ^from including:						
Trend^b^ (1 d.f.)	324***	4	50**	612***	1	185**
Continent^c ^(3 d.f.)	687***	47*	127***	2283***	205***	832***
Trend given continent (1 d.f.)	81***	1	30**	57**	27**	18(*)
Continent given trend (3 d.f.)	445***	44*	107***	1729***	231***	665***
Residual deviance^d ^(d.f.)	756 (124)	148 (38)	97 (38)	426 (86)	87 (33)	190 (33)
Deviance explained	50.4%	24.5%	61.8%	84.6%	72.7%	81.7%
Means (SEs)^d^:						
Constant	4.24 (0.18)	3.74 (0.49)	2.89 (0.43)	4.10 (0.12)	3.77 (0.28)	3.59 (0.31)
Trend	0.15 (0.04)	0.04 (0.11)	0.35 (0.10)	0.09 (0.03)	-0.21 (0.07)	0.14 (0.08)
Asia (not China)	-0.97 (0.18)	-1.44 (0.63)	-0.35 (0.31)	-0.70 (0.08)	-1.28 (0.29)	-0.49 (0.15)
Europe	-0.42 (0.12)	-0.93 (0.35)	-1.33 (0.26)	-1.04 (0.08)	-0.85 (0.19)	-1.32 (0.21)
America	-0.73 (0.10)	-0.83 (0.27)	-1.08 (0.19)	-1.12 (0.07)	-1.38 (0.15)	-1.27 (0.12)
Drop in deviance and p value^a ^from adding:^e^						
10 level region^f ^(6 d.f.)	103**	37	21	43	28(*)	21
5 level period^g ^(3 d.f.)	111***	34*	12	11	10	19
Interactions of trend and continent (3 d.f.)	22	7	15	55	9	7

^a ^p values are coded as *** p < 0.001, ** p < 0.01, * p < 0.05, (*) p < 0.1. If no code is shown p ≥ 0.1. d.f. = degrees of freedom.

^b ^Linear trend in period scoring 1 = 1930–60, 2 = 1961–70, 3 = 1971–80, 4 = 1981–90, 5 = 1991–99.

^c ^Continent defined as China, Asia (not China), Europe or America.

^d ^For model including linear trend in period and continent. Note that estimates for Asia (not China), Europe and America are relative to rates for China.

^e ^Drop in deviance from adding the specific variable to the model including linear trend in period and continent.

^f ^Levels as shown in Tables 10, 11, 12 and 13.

^g ^This tests for departure from linear trend.

For all lung cancer and for adeno, much of the variability associated with the trend in rate over period can be explained by adjustment for continent, the timing of the studies varying by continent. Nevertheless evidence remains of an increase in the rates over time in each sex for both endpoints. For squamous, no trend is evident in males, and in females adjustment for continent made the estimate negative (-0.21, SE 0.07).

The percentage of the deviance explained by the two factor model in continent and trend varied between analyses, from over 80% for all lung cancer and for adeno in females, to under 25% for squamous in males. There is no evidence of interaction between the trend and continent effects for any analysis, and in most of the analyses there is no evidence that introducing a 10 level region variable or a 5 level period variable adds significantly to the model. The main exception is for all lung cancer in males. Examination of the estimates (not shown) showed that this was caused by variation within Europe (high rates in the UK, low in Scandinavia and intermediate elsewhere), and the tendency for rates to be low in 1930–60 and higher in the other periods with no clear trend between 1961 and 1999.

Table 15 presents results of inverse-variance weighted regression analyses for ever smokers. All six analyses show strong evidence (p < 0.001) of an increasing trend after adjustment for continent. Although, for all the analyses for males and for all lung cancer for females, there is still evidence (p < 0.01 or p < 0.001) of variation by period given the trend, the additional deviance explained per degree of freedom by the linear variable is always substantially greater than that explained by the departure from trend. For all lung cancer in males, where the departure is most evident, it is caused by the estimated rate rising steeply from 1930–60 to 1961–70, then more slowly to 1981–90 and then falling somewhat.

**Table 15:** Inverse-variance weighted regression analyses – ever smokers

	**Males**			**Females**		
	**All**	**Squamous**	**Adeno**	**All**	**Squamous**	**Adeno**
Overall deviance (d.f.)	42483 (128)	3257 (42)	2618 (42)	6005 (90)	644 (37)	1228 (37)
Drop in deviance (d.f.) and associated p value^a ^from including:						
Trend^b ^(1 d.f.)	14470***	927***	1257***	2425***	205***	602***
Continent^c ^(3 d.f.)	6245***	454	594*	914**	38	224(*)
Trend given continent (1 d.f.)	17056***	1117***	1169***	2278***	196***	514***
Continent given trend (3 d.f.)	8831***	644**	505***	767***	29	136*
Residual deviance^d ^(d.f.)	19181 (124)	1687 (38)	855 (38)	2813 (86)	410 (33)	490 (33)
Deviance explained	54.9%	48.2%	67.3%	53.2%	36.3%	60.1%
Means (SEs)^d^:						
Constant	4.80 (0.16)	4.57 (0.30)	2.36 (0.41)	3.58 (0.27)	3.12 (0.41)	1.20 (0.60)
Trend	0.27 (0.03)	0.21 (0.04)	0.62 (0.09)	0.51 (0.06)	0.37 (0.09)	0.84 (0.14)
Asia (not China)	-0.22 (0.18)	-0.51 (0.33)	-0.14 (0.30)	-0.58 (0.34)	-0.72 (0.49)	-0.52 (0.49)
Europe	0.67 (0.14)	0.31 (0.28)	-0.44 (0.29)	-0.37 (0.17)	-0.14 (0.29)	-0.68 (0.39)
America	0.44 (0.14)	0.23 (0.27)	0.31 (0.25)	0.14 (0.14)	-0.19 (0.22)	0.18 (0.24)
Drop in deviance and p value^a ^from adding:^e^						
10 level region^f ^(6 d.f.)	6944***	368	336**	620**	114	84
5 level period^g ^(3 d.f.)	9002***	666***	279**	477**	54	15
Interactions of trend and continent (3 d.f.)	3825***	78	46	603***	84(*)	80

^a ^p values are coded as *** p < 0.001, ** p < 0.01, * p < 0.05, (*) p < 0.1. If no code is shown p ≥ 0.1. d.f. = degrees of freedom.

^b ^Linear trend in period scoring 1 = 1930–60, 2 = 1961–70, 3 = 1971–80, 4 = 1981–90, 5 = 1991–99.

^c ^Continent defined as China, Asia (not China), Europe or America.

^d ^For model including linear trend in period and continent. Note that estimates for Asia (not China), Europe and America are relative to rates for China.

^e ^Drop in deviance from adding the specific variable to the model including linear trend in period and continent.

^f^ Levels as shown in Tables 10, 11, 12 and 13.

^g ^This tests for departure from linear trend.

There is clear evidence (p < 0.01 or p < 0.001) of variation by continent after adjustment for linear trend for all the analyses for males and for all lung cancer for females. In most of these analyses there is also additional evidence of variation by region within continent. For all lung cancer, summarizing the findings simply is made more difficult by the evidence (p < 0.001) of an interaction between trend and continent, with, in each sex, the slope of the increase greater in America than in Europe. However, the analyses confirm the observation made earlier that, whereas for never smokers rates were consistently higher in China, this is not so for ever smokers.

Table 16 presents results of inverse-weighted regression analyses for current and former smokers for all lung cancer. There are too few sex-specific estimates for squamous and adeno to justify further analyses. Although there is no marked evidence of a trend for former smokers in females, the other analyses show a clear effect (p < 0.001). In males, there is also evidence of departure from trend for both current and former smokers with the rates rising up to 1981–90 and then falling as noted for ever smokers.

**Table 16:** Further inverse-variance weighted regression analyses for all lung cancer

	**Current smokers**		**Former smokers**	
	**Males**	**Females**	**Males**	**Females**
Overall deviance (d.f.)	14399 (69)	2926 (45)	2523 (69)	611 (45)
Drop in deviance (d.f.) and associated p value^a ^from including:				
Trend^b ^(1 d.f.)	5136***	1490***	485***	56*
Continent^c ^(3 d.f.)	2981**	989***	173	148**
Trend given continent (1 d.f.)	6544***	928***	491***	33(*)
Continent given trend (3 d.f.)	4389***	427**	178	126*
Residual deviance^d ^(d.f.)	4875 (65)	1009 (41)	1860 (65)	430 (41)
Deviance explained	66.1%	65.5%	26.3%	29.6%
Means (SEs)^d^:				
Constant	4.43 (0.41)	3.18 (0.64)	5.02 (0.65)	4.68 (0.85)
Trend	0.36 (0.04)	0.57 (0.09)	0.21 (0.05)	0.26 (0.15)
Asia (not China)	-0.13 (0.41)	-0.37 (0.62)	-0.47 (0.67)	-0.79 (0.80)
Europe	1.09 (0.39)	0.01 (0.56)	0.02 (0.62)	-1.49 (0.67)
America	0.80 (0.39)	0.61 (0.53)	0.16 (0.62)	-0.52 (0.61)
Drop in deviance and p value^a ^from adding:^e^				
10 level region^f ^(6 d.f.)	2274***	237	539**	55
5 level period^g ^(3 d.f.)	1658**	181(*)	647***	34
Interactions of trend and continent (3 d.f.)	370(*)	402***	111	46

^a ^p values are coded as *** p < 0.001, ** p < 0.01, * p < 0.05, (*) p < 0.1. If no code is shown p ≥ 0.1. d.f. = degrees of freedom.

^b ^Linear trend in period scoring 1 = 1930–60, 2 = 1961–70, 3 = 1971–80, 4 = 1981–90, 5 = 1991–99.

^c ^Continent defined as China, Asia (not China), Europe or America.

^d ^For model including linear trend in period and continent. Note that estimates for Asia (not China), Europe and America are relative to rates for China.

^e ^Drop in deviance from adding the specific variable to the model including linear trend in period and continent.

^f ^Levels as shown in Tables 10, 11, 12 and 13.

^g ^This tests for departure from linear trend.

Evidence of a variation by continent (given trend) is strongest for current smokers in males, where rates were clearly higher in Europe and America than in Asia. However, there is also variation by region within continent (p < 0.001), with rates higher in North than in South America, and in the UK and Eastern Europe than in Scandinavia or Western Europe. For current smoking females, rates are highest in America and there is no evidence of a variation by region within continent. While there is less evidence of regional variation in former smokers, it is interesting to note that, in males, region, but not continent, explained significant (p < 0.01) variation, with estimates highest for UK and Eastern Europe and lowest for Scandinavia and Other Asia.

Although for current smokers, the model including trend and continent explains 66% of the deviance (in both males and females), there is still evidence of interaction for females (p < 0.001), due to more sharply rising trends in America than elsewhere. For former smokers, the proportion of deviance explained is much less (26% males, 30% females) and there is no evidence of interaction.

## Discussion

### Never smoker rates

Our results clearly show that lung cancer rates in never smokers are markedly higher in China than in other regions studied. The excess is evident for all lung cancer and for squamous and adeno. One reason for this may be the common household use of poorly-vented stoves in various regions of China. It is interesting to note that estimates of global mortality attributable to smoking in 2000 published by Ezzati and Lopez in 2003 [19] take account of variation in the never smoker lung cancer rate based on household poorly-vented stove use. They cite evidence of substantial variations in never smoker lung cancer rates in China as being “largely a result of patterns of household energy use in China over the past decades” with “coal, a common household fuel in China and traditionally burned in stoves and buildings with poor ventilation.”

Our results also suggest some tendency for never smoker overall lung cancer rates to increase over time. The literature on this issue is not very consistent. Thus, while no evidence of a trend was seen comparing rates in the American Cancer Society CPS I and CPS II studies conducted about 20 years apart [4,20], or comparing rates by time of follow-up in the US Veterans study [5] or British Doctors study [6], there have been a number of reports of an increase in Japan [7,21], Sweden [8], Italy [22], the UK [23] or the USA [24,25], though some of the reports suggesting large increases tend to have clear technical weaknesses and be difficult to interpret [26]. Any time trend that does exist seems, from our analyses, to be more evident for adeno than for squamous. As mentioned later, when we consider the limitations of our indirect method for estimating lung cancer risks by smoking habit, there is evidence that this may be associated with changes over time in categorization of lung cancer type at diagnosis.

Our results also show some excess of never smoking lung cancer rates in males for all lung cancer and for squamous. Although we have excluded estimates from studies specifically in occupationally exposed groups, this excess may still be associated with increased exposure to occupational exposure to carcinogens in males.

### Ever smoking rates

The excess in rates for males is more evident for ever smokers than for never smokers. This is unsurprising in view of the higher prevalence of smokers in males, their greater daily cigarette consumption, and their earlier take up of the habit.

The pattern of variation by region is also very different for ever smokers and for never smokers. While this clearly depends on between-regional differences in aspects of smoking such as prevalence, intensity, duration, extent of quitting and type of product smoked, it also reflects the substantially lower relative risk for ever smokers in Asia highlighted in our first report on the IESLC database [3]. Whereas estimated rates for never smokers in China are much higher than in other regions, each of the analyses conducted for ever smoking (by sex and endpoint) give estimates that are higher than China for a number of regions of Europe and North America. Rates for ever smokers for all lung cancer and for squamous seem rather lower in Scandinavia, Japan, and in parts of Asia other than China or Japan.

The tendency for rates to increase with time is also more evident for ever smokers than for never smokers, and is particularly evident for adeno. The observation that rates for adenocarcinoma have risen relative to those for squamous cell carcinoma has been made a number of times in the literature, the suggestion often being made [16,27,28] that this is due to changes in the design of cigarettes. Though this may not be the explanation, inasmuch as there is no evidence of an increased risk of adenocarcinoma associated with tar reduction or the switch from filter to plain cigarettes [3,29], our results do indeed suggest that adeno forms an increasingly large part of overall lung cancer rates over time.

### Current and former smokers

Many of the conclusions follow, not unexpectedly, the results for ever smokers. Thus, for both current and former smokers, rates are higher in males, and there is evidence of an increase in rates over time. The pattern of variation by continent for current smokers is also not dissimilar from that for ever smokers, with rates highest in Europe and America for males, and in America for females. As for ever smoking males, current smoking males also show evidence of departure from trend and of interaction between trend and continent, making it difficult to describe the patterns succinctly. For former smokers, continent and period explain less of the deviance than for current smokers. This is likely to be partly due to the smaller relative risks for former than current smokers, and the fact that the analyses do not take account of mean time of quit which will vary by continent (as the timing of the anti-smoking message was later in Asia than in Europe or America), and by year (as long-term quitters would have been less common earlier on).

### Limitations

When considering the results presented, there are a number of limitations that should be borne in mind. Considering first the lung cancer mortality data extracted from the WHO database, one should note that it is only available for all lung cancer and not by histological type, and that diagnosis may be inaccurate, with misdiagnosis rates varying by country and time [30]. Although the definition of lung cancer under the various revisions of the ICD relevant to this report are essentially unchanged, coding practices may have varied. Excessive use of codes for ill-defined and unknown causes and incomplete death registration coverage may have detracted from the quality of the data, with only 33% of relevant countries recently assessed as providing “high quality” data [31]. For some countries, data relate only to selected regions (Table 1), with data for China derived from a sample registration scheme including less than 10% of all deaths occurring in the country [1].

Furthermore, though survival rates remain very poor, trends in mortality may not necessarily reflect trends in disease incidence. Cancer incidence rates are available, but for a far narrower range of countries and time periods.

There are also a number of limitations with the data on relative risk by smoking habit obtained from the IESLC database. These include variations in definition of smoking, definition of disease and extent of adjustment for confounders, and bias due to misclassification of smoking status. These and some other issues are also discussed in the first paper on IESLC [3], but some of the principal points are considered below.

As regards definition of smoking, relative risks were selected for smoking of any product, if available, and of cigarettes (or cigarettes only) otherwise. In countries where pipe and cigar smoking is rare, this distinction may be of little consequence, but it may be more important in some countries. The type of cigarette smoked is also relevant, and though no clear difference in risk has been noted between the flue-cured cigarettes smoked in the UK and various other (mainly Commonwealth) countries [2,32] or between mentholated and unmentholated cigarettes [33], there is clear evidence that risk is greater in handrolled than manufactured cigarettes [29], in black than blond tobacco cigarettes [34], and in higher tar plain cigarettes than in lower tar filter cigarettes [35].

As can be seen in Table 3, variation exists in the definition of all lung cancer, squamous and adeno. While for the great majority of studies the definitions include, respectively, all cases, only cases of squamous cell carcinoma, and only cases of adenocarcinoma, in a small number of studies alternative definitions were allowed. Thus, for all lung cancer our definitions also includes (i) all cases other than alveolar cell cancer, (ii) all cases except lung cancers of mixed cell types, (iii) only cases of squamous cell carcinoma and adenocarcinoma, (iv) as definition (iii) but also small cell carcinoma, and (v) as definition (iv) but also large cell carcinoma. Definitions of “squamous” also included (i) Kreyberg I lung cancers, (ii) all lung cancers except adenocarcinoma, and (iii) squamous cell and differentiated carcinomas and (iv) squamous cell and small cell carcinomas. Definitions of “adeno” also include Kreyberg II lung cancers, (ii) adenocarcinomas and large cell carcinomas, (iii) all lung cancers except squamous cell and undifferentiated carcinomas, and (iv) all lung cancers except squamous cell and small cell carcinomas. While it would have been possible to make the data “purer” by omitting such alternative definitions (and also only allowing data for smoking of any product), this would have reduced the number of studies available, and lost power.

A related issue is change over time in the diagnosis of lung cancer types. Though it is generally recognized that the relative frequency of adenocarcinoma to squamous cell carcinoma has changed over time (e.g. [16,36]), there are reports [37,38] of studies which re-evaluated diagnoses conducted in previous years, finding that many lung cancers initially considered to be squamous cell carcinomas should, according to more modern criteria, be considered adenocarcinomas.

Although we preferred to use unadjusted relative risks as being directly relevant to the national mortality rate, we did include adjusted relative risks for squamous and adeno due to the scarcity of unadjusted data. This is unlikely to have had any major effect as we previously demonstrated that adjustment had little effect on the relative risks [3].

The issue of misclassification of smoking status is perhaps more serious. Some years ago, we carried out extensive work on the misclassification of smoking status and the effect it has in biasing the estimates of the association between environmental tobacco smoke exposure and lung cancer [39-42]. For many of our calculations we assumed that, in Western populations, the bias may be equivalent to that caused by 2.5% of average lung cancer risk ever smokers reporting that they have never smoked. For Asian populations, the percentage is clearly higher (see e.g. [43]), perhaps 10% or 20%. If these rates apply, and there are considerable uncertainties [39,44], misclassification will have a marked effect on the estimated lung cancer death rates in never smokers.

To illustrate this, consider a population in which 50% have ever smoked, and in which the true relative risk for ever vs never smoking is 8. Suppose also that the overall lung cancer death rate is 45. Based on these “true” data, the indirect estimates of rates by our method would be 10 in never smokers and 80 in ever smokers. If in fact 2.5% of ever smokers are misclassified as never smokers, one can then readily show that one will observe 48.75% to have smoked, and a relative risk of 6.83. Based on the “observed” data, the estimated rates will then still be 80 in ever smokers but will be 11.7, not 10, in never smokers. For misclassification rates of 10% and 20%, the estimated rates in never smokers will be higher still, respectively, 16.4 and 21.7, corresponding to “observed” relative risks of 4.89 and 3.69. The extent of the bias increases, not only with the misclassification rate, but also with the true proportion of ever smokers.

Other limitations concern combining the relative risk data from IESLC with the national rates from WHO. One relates to the fact that most of the relative risk estimates derive from studies that are not nationally representative but are drawn from populations of a variety of types. We have sought to minimize this problem by excluding studies conducted in populations that were grossly unrepresentative, as described in the Methods section. Relative risks based on a variety of populations are frequently subject to meta-analysis in an attempt to get an overall average risk which can be taken to apply generally, and our use of relative risks derived from somewhat unrepresentative populations involves essentially the same underlying assumption.

Lack of national representativeness of the IESLC study populations will also mean that the estimated distribution of smoking habits may not be the same as that seen in the country where the study was conducted. If the at risk population in a cohort study (or the control population in a case-control study) contains too low a proportion of ever smokers, national rates in both ever and never smokers will be overestimated, and if it contains too high a proportion they will be underestimated. For example, assuming that the relative risk is 9, the national lung cancer rate is 100 and the national population actually contains 50% ever smokers, the true rates of 20 in never smokers and 180 in ever smokers will be estimated as 23.8 and 214.3 if the control/at-risk population contains 40% ever smokers, and as 17.2 and 155.2 if the population contains 60% ever smokers. Such biases seem unlikely to affect our conclusions, as they seem much smaller than the marked differences seen by region and period. In any case it is unclear why such biases should cause spurious regional differences or trends.

Another issue relates to which WHO 5 year period data to use for a given study. For case-control studies we use the midpoint year of the interviews, while for prospective studies, we use a survival-adjusted mid-point of the follow-up period. Although both are open to question, this is unlikely to cause any major error. Nor is the use of substitute years (see Table 1). The need for this was relatively rare, and sometimes involved only quite small differences in time.

A major feature of our methodology is that it applies all age relative risks from studies based on populations of varying ages to estimate lung cancer rates by smoking habit for age 70–74, based on overall WHO rates for that age group. This issue is discussed in the Methods section “Testing the validity of the method with respect to age”. This gives justification for our decision to select age 70–74 rather than any other age range, and points out that studies of young populations were excluded from consideration. It should also be noted that age-specific data on lung cancer relative risks are very limited, and even then are not for five year age groups. Any weaknesses resulting from the decision to use age 70–74 rates seem likely to apply similarly in the various studies considered, and should therefore not affect conclusions regarding variations by sex, region and time period.

We should also point out that our meta-regressions are relatively limited. Better understanding of patterns in rates over time and region may be gained by additional analyses which take into account aspects of the studies used to generate the rates. The relevant data for others to attempt this are available from the Tables in this report and from our original paper based on the IELSC database [3].

## Conclusions

Data on lung cancer mortality rates by smoking habit are not available nationally, and studies presenting estimates are quite limited in scope, particularly for current and former smokers, and by histological type. This deficiency can hinder interpretation of the evidence on factors associated with lung cancer risk, a deficiency we have tried to rectify using an indirect estimation method. Estimates of absolute rates by country, sex, smoking habit and histological type were derived from 148 epidemiological studies by linking their findings to WHO national lung cancer mortality data. There are a number of potential limitations of the method, due to such factors as variations in definition of smoking and lung cancer type in the epidemiological database, changes over time in diagnosis of lung cancer types, lack of national representativeness of some studies, and regional variation in smoking misclassification rates. However many features of the results are consistent with the epidemiological literature. These include the high never smoker lung cancer rates in China, the increasing trend in rates over time in smokers, and the tendency for adeno rates to rise relative to squamous rates. This gives some confidence in the results, and suggests that other conclusions to be drawn from the indirect rates have validity. For example, the observation that, over the period 1930–2000, estimated rates for adeno among never smokers have risen markedly compared to the corresponding rates for squamous, strongly suggests that changes in the type of cigarette smoked are unlikely to explain the marked increase in the relative frequency of adenocarcinoma to squamous cell carcinoma.

## Abbreviations

CI: Confidence interval; CPSI: American cancer society cancer prevention study I; IESLC: International epidemiological studies on smoking and lung cancer database, described in detail elsewhere [3]; SE: Standard error.

## Competing interests

PNL, founder of P.N.Lee Statistics and Computing Ltd., is an independent consultant in statistics and an advisor in the fields of epidemiology and toxicology to a number of tobacco, pharmaceutical and chemical companies. This includes Philip Morris Products S.A., the sponsor of this study. BAF is an employee of P.N.Lee Statistics and Computing Ltd.

## Authors’ contributions

PNL and BAF were responsible for planning the study. The statistical analyses were conducted by BAF along lines discussed and agreed with PNL. PNL drafted the paper, which was then critically reviewed by BAF. Both authors read and approved the final manuscript.

## Pre-publication history

The pre-publication history for this paper can be accessed here:

http://www.biomedcentral.com/1471-2407/13/189/prepub

## Supplementary Material

Additional file 1 **Rejected studies. **This file lists the studies from the original IESLC database which were rejected from the current work, with reasons for rejection. Click here for file
