# Supplementary material for: Indirectly estimated absolute lung cancer mortality rates by smoking status and histological type based on a systematic review
Source: BMC Cancer. 2013 Apr 9;13:189. doi: 10.1186/1471-2407-13-189 (PMC3639928; doi:10.1186/1471-2407-13-189)
Supplement: Additional file 1 — Rejected studies. This file lists the studies from the original IESLC database which were rejected from the current work, with reasons for rejection. [file 1471-2407-13-189-S1.doc]

# Indirectly estimated absolute lung cancer mortality rates by smoking status and histological type based on a systematic review

Peter N Lee and Barbara A Forey

# Additional file 1 – Rejected studies

Studies were taken from the IESLC database, and the corresponding references and a brief description for each study are given in Table 1 of reference [3].

Reasons for rejection are listed below. For each rejected study, only the first reason is given. The reason given may be relevant only to relative risks (RRs) remaining after earlier rejections (e.g. for study CORREA, once combined-sex RRs [which had confidence intervals] were rejected, only RRs which lacked a confidence interval remained).

## No relative risks available for ever vs never smokers

BLOT1, BLOT3, BOUCHA, BUELL, BYERS2, ENSTRO, GUO, HIRAY2, KANELL, LAURIL, LIAW, LICKIN, MOLLO, MRFIT, MURATA, MZILEN, NOTANI, RESTRE, RIMING, SAARIK, SEGI2, SHIMIZ, SITAS, SOBUE2, TANG2, WALD, WARSIN, WATSON, WU2, WYNDE5, WYNDE8, WYNDER

## Only combined-sexes results available

AUSTIN, AUVINE, AXELSO, CASCO2, CASCOR, CHATZI, CHEN, CHEN3, COOKSO, DOCKER, GARCIA, GARDIN, GER, HEGMAN, HUANG, ISHIMA, KAUFMA, KOHLME, LEMARC, LETOUR, LIU, LIU5, LUO, ODRISC, PERSH2, PISANI, POFFIJ, POLEDN, RADZIK, ROOTS, ROTHSC, SHAW, SPITZ, STOCKW, SUN, SUZUK2, TANG, ULMER, VEIERO, WANG2, WANG3, XU2, XU4, YAMAGU, YONG

## Age range unknown

BERRIN, DEKLER, REN, ZHENG

## Multi-country study

BOFFET, LUBIN2

## Country lacking relevant WHO mortality data

DEAN, DOSEME, HAMMO2, JUSSAW, NOTAN2, PARKIN, SANKAR

## Confidence intervals for relative risks not available

CORREA, TAO

## Results available only for menthol cigarette smoking

CARPEN

## Results available only for adenocarcinoma

BROWN1, SUZUKI

## Subsidiary study (results from corresponding principal study included)

PRESCO, QIAO, WYNDE7

## Age range below 60

HEIN, KINLEN, SPEIZE, TSUGAN, TVERDA, WANG4

## Minority racial group

CHYOU, HINDS

## Risky occupation

AMANDU, AMES, ARCHER, CHIAZZ, DUNN, GARSHI, HANSEN, JARUP, JARVHO, LAUSSM, LIDDEL, LUBIN, MAGNUS, MARSH, MARSH2, MARTIS, MASTRA, MCLAUG, QIAO2, TOKARS, WICKLU, XIANGZ

## Other non-general population

AKIBA (atomic bomb survivors), MRFITR (high coronary risk)

## Only adjusted RRs available for AllLC[[1]](#footnote-2)

CEDERL, GODLEY, HUMBLE, KAISER, KO, LEVIN, MILLS, YUAN, ZHANG

# References

See main paper.

1. Note however that age-adjusted results from prospective studies are included [↑](#footnote-ref-2)
